# Bright Quantum-Grade Fluorescent Nanodiamonds

**DOI:** 10.1021/acsnano.4c03424

**Published:** 2024-12-16

**Authors:** Keisuke Oshimi, Hitoshi Ishiwata, Hiromu Nakashima, Sara Mandić, Hina Kobayashi, Minori Teramoto, Hirokazu Tsuji, Yoshiki Nishibayashi, Yutaka Shikano, Toshu An, Masazumi Fujiwara

**Affiliations:** † Department of Chemistry, Graduate School of Life, Environmental, Natural Science and Technology, Okayama University, Okayama 700-8530, Japan; ‡ The National Institutes for Quantum Science and Technology (QST), Institute for Quantum Life Science (iQLS), Chiba 263-8555, Japan; § Advanced Materials Laboratory, Sumitomo Electric Industries, Ltd., Hyogo 664-0016, Japan; ∥ Institute of Systems and Information Engineering, University of Tsukuba, Tsukuba, Ibaraki 305-8573, Japan; ⊥ Center for Artificial Intelligence Research (C-AIR), University of Tsukuba, Tsukuba, Ibaraki 305-8577, Japan; # Institute for Quantum Studies, Chapman University, Orange, California 92866, United States; ∇ School of Materials Science, Japan Advanced Institute of Science and Technology, Nomi, Ishikawa 923-1292, Japan

**Keywords:** nanodiamonds, nitrogen-vacancy centers, spins, spin-relaxation times, quantum biosensor, cellular probes

## Abstract

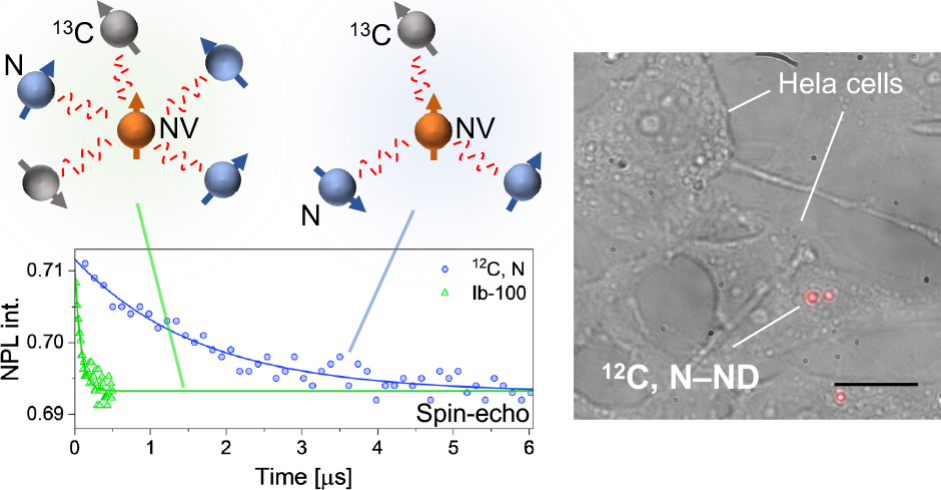

Optically accessible spin-active nanomaterials are promising as quantum nanosensors for probing biological samples. However, achieving bioimaging-level brightness and high-quality spin properties for these materials is challenging and hinders their application in quantum biosensing. Here, we demonstrate bright fluorescent nanodiamonds (NDs) containing 0.6–1.3-ppm negatively charged nitrogen-vacancy (NV) centers by spin-environment engineering via enriching spin-less ^12^C-carbon isotopes and reducing substitutional nitrogen spin impurities. The NDs, readily introduced into cultured cells, exhibited improved optically detected magnetic resonance (ODMR) spectra; peak splitting (*E*) was reduced by 2–3 MHz, and microwave excitation power required was 20 times lower to achieve a 3% ODMR contrast, comparable to that of conventional type-Ib NDs. They show average spin-relaxation times of *T*_1_ = 0.68 ms and *T*_2_ = 3.2 μs (1.6 ms and 5.4 μs maximum) that were 5- and 11-fold longer than those of type-Ib, respectively. Additionally, the extended *T*_2_ relaxation times of these NDs enable shot-noise-limited temperature measurements with a sensitivity of approximately . The combination of bulk-like NV spin properties and enhanced fluorescence significantly improves the sensitivity of ND-based quantum sensors for biological applications.

## Introduction

Engineering spin-active materials is critical to developing highly sensitive quantum nanosensors, as demonstrated by nanodiamonds (NDs) containing color defect centers,^1,2^ organic nanosolids with radical molecules,^3^ rare earth nanocrystals,^4^ and nanoflakes of hexagonal boron nitrides.^5,6^ Among these spin-active materials, NDs containing nitrogen-vacancy (NV) centers are the most advanced quantum nanosensors utilized for biological applications owing to their multimodal sensing capability,^7,8^ photostability,^9^ chemical functionality,^10^ and biocompatibility.^11^ The NV quantum nanosensors exploit the dependence of optically accessible NV electron spins on magnetic field, electric field, and temperature of the surrounding cellular environment,^12^ which enables subcellular measurements of either those values or extended physicochemical parameters, such as pH,^13,14^ magnetic ions,^15,16^ reactive oxygen species^17,18^ and rheology.^19,20^ In the majority of the cases, these sensing modalities measure the frequency shift (or modulated relaxation times) in optically detected magnetic resonance (ODMR) of NV centers. Therefore, measurement sensitivity is critically dependent on the quality of the NV spin properties.^21^

**Figure 1:**
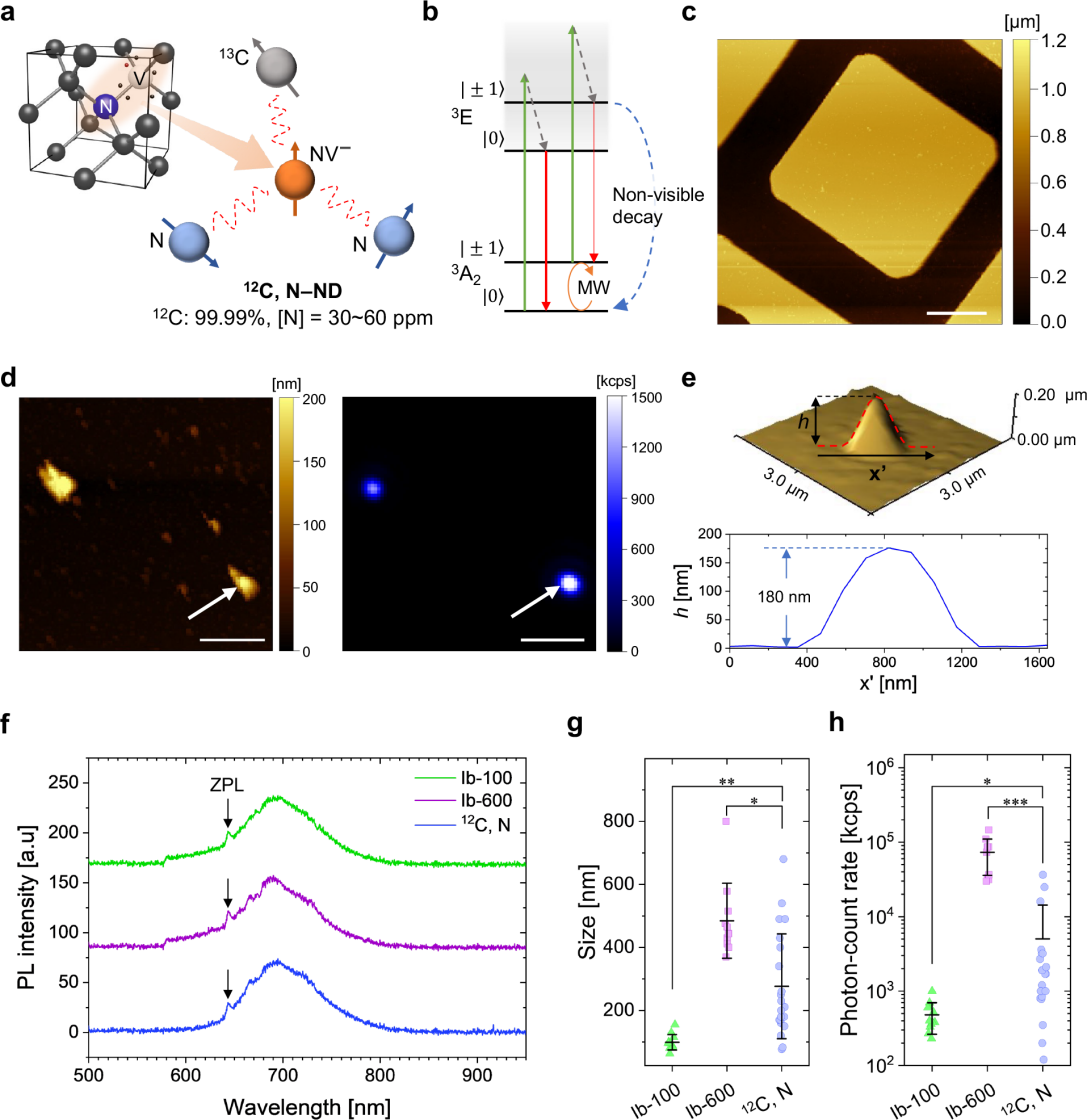
(a) Illustrations of the NV crystal structure and the interaction of NV with the spin bath of N and ^13^C. (b) Schematic representation of simplified energy level structure of NV centers. |0⟩ and |±1⟩ are the spin sublevels for *m*_s_ = 0 and *m*_s_ = ± 1, respectively. MW: microwave. ^3^A_2_ (^3^E): triplet ground (excited) state. (c) AFM topography image of a single grid engraved on a coverslip. Scale bar: 10 μm. (d) AFM topography and the corresponding confocal fluorescence images of ^12^C, N-NDs on a grid. Scale bars: 2 μm. (e) Three-dimensional visualization of the topography of the ND indicated by the white arrow in Figure 1d (top) with a cross-section along the *x*′ axis (bottom). (f) Fluorescent spectra of type-Ib NDs and ^12^C, N-NDs. (g) Statistical plots of the ND size (the ND height (*h*) in Figure 1e) determined by AFM and (h) photon-count rate at an optical excitation intensity of ∼7 kW cm^–2^ for Ib-100, Ib-600, and ^12^C, N-NDs. Mean and standard deviation (1σ) are indicated in the statistical plots. The error bar is shown only for the upper error side (+σ) for ^12^C, N-NDs in Figure 1h, where a large standard deviation (σ = 5000 kcps) makes a negative lower side (−σ) invisible in a log plot. Statistical significance is indicated as follows: *p < 0.05, **p < 0.01, ***p < 0.001.

However, current fluorescent NDs incorporating high-density NVs that show bioimaging-level brightness exhibit deficient spin qualities in contrast to bulk diamonds. They show broad ODMR spectra and short spin relaxation times, which substantially deteriorates the measurement sensitivity.^21,22^ The NV spins are affected by (i) a high concentration of spin impurities (Figure 1a) and (ii) surface spin noise.^23−25^ However, recent studies suggest that the effects of these factors on NDs might be mitigated. First, high-yield NV production and dense NV ensembles have been realized in high-quality synthetic bulk diamonds. Furthermore, reduction of substitutional nitrogen impurity and enrichment in spineless ^12^C-carbon isotopes have been achieved, benefiting quantum sensing applications.^26−29^ These bulk diamonds exhibited long spin relaxation times and demonstrated applications, such as nanoscale nuclear magnetic resonance^30,31^ and neural monitoring.^7,32^ Second, previous studies revealed that the surface spin noises reduced longitudinal spin relaxation time (*T*_1_) of single NV spin in NDs at *d*^–4^ scale with *d* being the ND diameter.^33^ The spin deterioration of NVs is prominent only for *d* < 80 nm, and this surface effect might not be predominant for a majority of the NDs used in quantum biological applications owing to their relatively large diameter (*d* > 80 nm).^1,2,19,34^ This is corroborated by the fact that long coherence time (transverse relaxation time; *T*_2_) was measured for single NV in NDs wherein the spin impurity concentration was minimized down to ppb level, allowing quantum sensing applications.^24,35^

Here, we demonstrate NDs possessing bulk-like NV spin properties by controlling the spin impurities of ^13^C and N, while increasing the NV concentration for bioimaging-level brightness. The spin relaxation times of the NVs, i.e., *T*_1_ and *T*_2_, in these spin-controlled NDs are enhanced by a factor of 5 for *T*_1_ and 11 for *T*_2_ (*T*_1_ = 0.68 ± 0.48 ms, *T*_2_ = 3.2 ± 1.2 μs), compared with conventional type-Ib NDs. Furthermore, the study introduces a thermometry method, thermal echo (TE), achieving a shot noise limited sensitivity of approximately . The results indicate the possibility of realizing quantum-grade NDs that can implement various quantum-enhanced measurement protocols for biological samples and applications.

## Results

### Determination of Size and NV Concentration for ^12^C, N-NDs

To achieve a high NV concentration exhibiting bioimaging-level brightness with simultaneous reduction in the amount of spin impurities, we use single-crystalline bulk diamonds, wherein the major spin impurities of N (the so-called P_1_ center) and ^13^C are minimized, and that are further enriched in NV by electron beam irradiation followed by high temperature annealing (Figure 1a). We prepared NDs via pulverization of single-crystalline bulk diamonds grown using a high-pressure high-temperature (HPHT) method with controlled impurities of [^12^C] = 99.99% and [N] = 30–60 ppm (see Methods). These NDs (hereafter called ^12^C, N-NDs) exhibit NV red fluorescence under green optical excitation with ODMR signal upon microwave irradiation (Figure 1b). To characterize the fluorescence brightness and size, the NDs were suspended in water and drop-casted onto coverslips with engraved island grids (Figure 1c). We performed single-particle characterization using confocal fluorescence and atomic force microscopy (AFM). Figure 1d shows the topography and corresponding confocal fluorescence images of the area containing several ^12^C, N-NDs. We observe bright fluorescence emission from these NDs (with a photon-count rate of 1500 kcps) at an optical excitation intensity of ∼7 kW cm^–2^. Their mean fluorescence intensity is comparable to the type-Ib NDs, sufficient for bioimaging (Figure S1a–d). A close view of the ND topography indicated by the white arrow is shown in Figure 1e, where the size is determined to be *h* = 180 nm by taking the height of the sample from the xy-imaging plane. Further, Figure 1f shows the typical fluorescence spectra with zero phonon line at 637 nm, confirming that the fluorescence of the ^12^C, N-NDs primarily originates from the negatively charged NVs.

The concentration of negatively charged NV ([NV^–^]) in NDs is typically determined by electron paramagnetic resonance (EPR) spectroscopy.^36^ However, this method requires hundreds of milligram of samples and cannot be employed for the present NDs due to the small amount. Alternatively, we determined [NV^–^] of the NDs by measuring their size via AFM (Figure 1g) and fluorescence intensity (Figure 1h) and comparing them with those of two types of type-Ib NDs characterized by dynamic light scattering (DLS) with mean sizes of 100 nm (Ib-100) and 600 nm (Ib-600) in the supplier’s specification sheet. The size analysis by AFM revealed the ND mean sizes as 98.5, 485, and 277 nm for the Ib-100, Ib-600, and ^12^C, N-NDs, respectively (Figure 1g). These mean sizes, which were relatively smaller than the DLS sizes, reflected the platelet morphology of ND^37^ (see Supporting Information for further morphological analysis, Section S1). Because the number of NVs is proportional to the volume of NDs, we assume that the fluorescence intensity is proportional to the ND volume: *I* ∝ [NV^–^]*V*, where *I* and *V* are the fluorescence photon-count rate and volume of the NDs, respectively. To determine [NV^–^] of the ^12^C, N-NDs from [NV^–^] of Ib-100 and Ib-600, we used the relationships [NV^–^]_^12^C,N_ = *I*_^12^C,N_*I*_Ib-100_^–1^*V*_Ib-100_*V*_^12^C,N_^–1^[NV^–^]_Ib-100_ and [NV^–^]_^12^C,N_ = *I*_^12^C,N_*I*_Ib-600_^–1^*V*_Ib-600_*V*_^12^C,N_^–1^[NV^–^]_Ib-600_, respectively. [NV^–^] of the ^12^C, N-NDs was 0.6–1.3 ppm, which was determined based on [NV^–^] ≈ 3 and 3.5 ppm for the Ib-100 and for Ib-600 NDs, respectively; these [NV^–^] values of the Ib-100 and for Ib-600 NDs were adopted from the specification sheet and were considered based on the conceivable ND morphology in terms of the aspect ratio of the NDs (see Supporting Information, Section S1).

### NV Spin Characterization by Continuous-Wave ODMR

**Figure 2:**
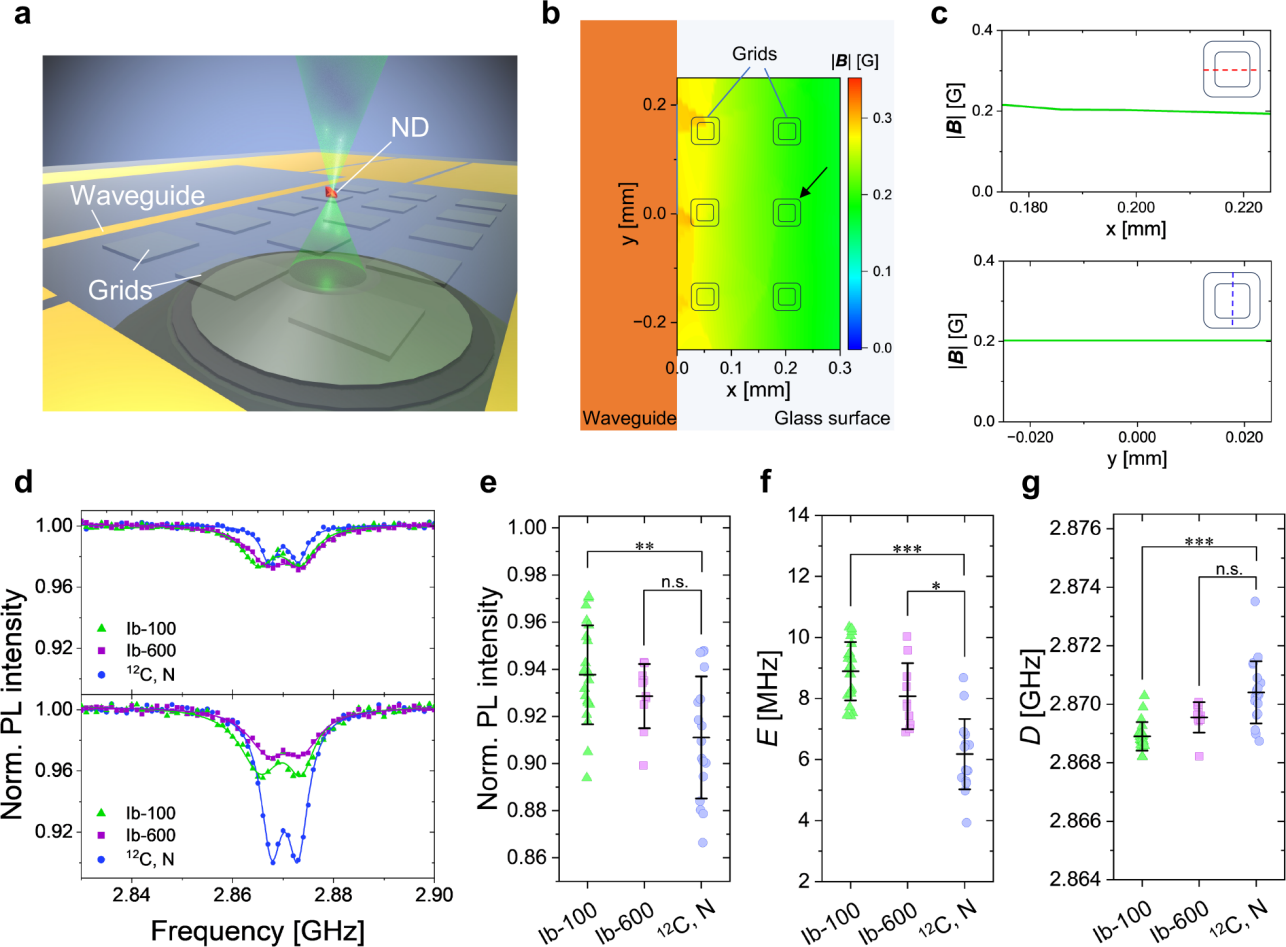
(a) Schematic representation of NDs on grids of the notch-shaped microwave antenna. (b) Heat map of simulated magnetic field (|**B**|) on the antenna overlaid with schematic grid structures. 26.1 mW microwave power was used for the simulation. (c) Cross sections of |**B**| over the grid indicated by a black arrow at (*x*, *y*) = (0.2, 0.0) mm along *x* (top) and *y* axes (bottom). (d) Representative CW-ODMR spectra of an Ib-100, Ib-600, and ^12^C, N-ND in the absence of an external magnetic field when the microwave power was adjusted to give 3% ODMR contrast (top) and with the identical microwave power of 26.1 mW at the input of the notch area (bottom). A identical optical intensity was used in both the panels (∼6 kW cm^–2^). The lines are double-Lorentzian fits. Statistical plots for the Ib-100 (green triangle), Ib-600 (purple square), and ^12^C, N-NDs (blue circle) for (e) ODMR depth, (f) *E*, and (g) *D*. Mean and standard deviation (1σ) are indicated in the statistical plots. Statistical significance is indicated as follows: *p < 0.05, **p < 0.01, ***p < 0.001.

The quantitative characterization of the continuous-wave (CW)-ODMR spectra over different NDs requires a well-defined spatial microwave-excitation-field pattern because the corresponding spectral shape is sensitive to the applied microwave intensity. Therefore, we exploited an on-chip platform based on a previously developed notch-shaped antenna pattern,^38^ which provided a uniform distribution of the microwave magnetic field (|**B**|) that was quantitatively defined at each grid (Figure 2a,b).

In this experiment, we selected the second nearest grid (*x* = 0.2 mm) from the edge of the central waveguide (*x* = 0.0 mm), where the variation in |**B**| was only 0.02 G over the area for the microwave power of 26.1 mW (14.2 dBm) utilized in the CW-ODMR experiments, as shown in Figure 2b,c (see Table S1). This variation resulted in only a 0.8% difference in the ODMR depth and a 10% change in the Rabi-frequency period, which enabled the characterization of the NV spins at the same microwave power without requiring Rabi frequency measurement for each ND. To observe the ODMR differences among the Ib-100, Ib-600, and ^12^C, N-NDs, the microwave power required to attain a 3% ODMR contrast was determined for each type of these NDs at the same optical intensity. The ^12^C, N-NDs exhibited a 3% ODMR contrast with a microwave power of 1.04 mW in the detection area (|**B**| = 0.041 G in the grid position), whereas the Ib-100 and Ib-600 NDs required 10.4 mW (|**B**| = 0.13 G) and 20.7 mW (|**B**| = 0.18 G) to obtain a 3% contrast (top panel in Figure 2d). This result indicates that, to attain the same 3% ODMR contrast, the microwave power required by the ^12^C, N-NDs is 10 and 20 times lower than those required by the Ib-100 and Ib-600 NDs, respectively. Moreover, the ODMR spectra of the ^12^C, N-NDs were substantially narrower than those of the type-Ib NDs. We determined the spectral evolution of all the NDs under an applied microwave power of 26.1 mW (bottom panel in Figure 2d). The ODMR contrast shown by the ^12^C, N-NDs was more than twice larger than those shown by the Ib-100 and Ib-600 NDs, and this result confirmed the improvement in the NV spin properties of the NDs developed in this study.

Subsequently, we performed a statistical analysis of the CW-ODMR spectra of these NDs (Figure 2e–g). The results demonstrated that, on average, the ODMR depth was greater for the ^12^C, N-NDs than for the Ib-100 and Ib-600 counterparts (Figure 2e). We further analyzed spectral parameters for peak splitting (*E*) related to crystal strains and zero-field splitting (*D*) to evaluate more NV intrinsic spin properties of the NDs (see Methods). The mean of *E* for the ^12^C, N-NDs was smaller than those for the type-Ib NDs by 2–3 MHz (Figure 2f), indicating reduced crystal strain in the ^12^C, N-NDs. The effect of the geomagnetic field on *E* was insignificant due to the random NV quantization axes (see Supporting Information, Section S4). Unexpectedly, the mean *D* of the ^12^C, N-NDs was 1–2 MHz higher than that of the Ib-100 NDs (Figure 2g). Given the mean *D* of Ib-600 was higher than that of Ib-100 as well, this increment in the mean *D* possibly results from the ND size difference among the ^12^C, N-and type-Ib NDs; however, the absolute value of *D* has not been studied in the context of ND size, and the exact origin of the increased *D* continues to remains unclear. Note that additional Raman measurements did not show the size dependency of the diamond peak (see Supporting Information, Section S5).

### NV Spin Characterization by Pulsed ODMR

**Figure 3:**
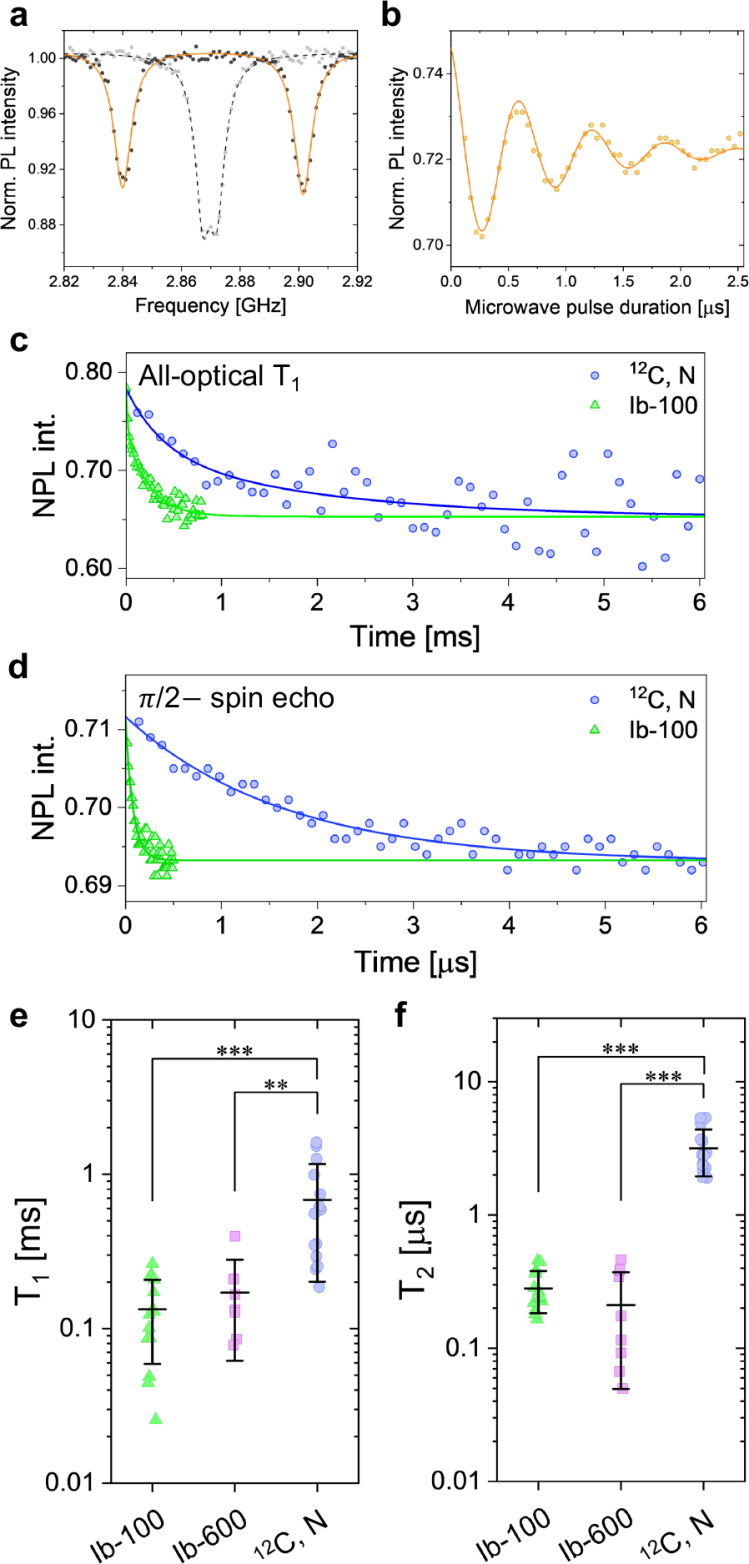
(a) ODMR spectra and double-Lorentzian fits of a representative ^12^C, N-ND with (black dots and orange solid line) and without (gray dots and gray dashed line) an external magnetic field. (b) A typical Rabi oscillation observed in the ^12^C, N-NDs. A solid line is a sine-damp fit. Representative profiles of (c) all-optical *T*_1_ relaxometry with decay times of 1.9 ms (^12^C, N) and 0.24 ms (Ib-100) and (d) π/2-spin echo measurements with the decay times of 1.7 μs (^12^C, N) and 0.079 μs (Ib-100). Statistical plots of *T*_1_ (e) and *T*_2_ (f) relaxation times for the ^12^C, N-, Ib-100 and Ib-600 NDs, respectively (*T*_1_^mean^ = 0.68 ± 0.48 ms, *T*_2_^mean^ = 3.2 ± 1.2 μs). Statistical significance is indicated as follows: “n.s.” denotes “not significant”, *p < 0.05, **p < 0.01, ***p < 0.001.

To evaluate the intrinsic NV spin properties of ^12^C, N-NDs, we performed pulsed-ODMR experiments and determined the *T*_1_ and *T*_2_ relaxation times of ^12^C, N-, and Ib-100 NDs (Figure 3a–d). An external magnetic field was applied in a controlled orientation to split the spectra into two peaks (Figure 3a). By addressing the lower-energy peak, we measured Rabi nutation to determine the duration of π-pulse used in the subsequent *T*_1_ and *T*_2_ measurements, where the typical duration of the π-pulse is 300–400 ns (Figure 3b). Figure 3c,d show the representative *T*_1_ and *T*_2_ profiles for the ^12^C, N- and Ib-100. Both profiles exhibit a substantial extension of the relaxation times in the ^12^C, N-NDs. *T*_1_ profiles were fit with a biexponential decay, and *T*_2_ profiles with a stretched exponential decay (see Methods). We performed statistical analysis by measuring a number of NDs as shown in Figure 3e,f. The ^12^C, N-NDs exhibit *T*_1_^max^ = 1.6 ms and *T*_2_^max^ = 5.4 μs in maximum, which are close to the bulk-limited relaxation times for the present nitrogen concentration of 30–60 ppm (*T*_1_ ≈ 3 ms,^39^*T*_2_ ≈ 3–5 μs^28^). *T*_1_ of the ^12^C, N-NDs (*T*_1_^mean^ = 0.68 ± 0.48 ms) was more than 4–5 times longer than those of the type-Ib NDs (Ib-100, 0.13 ± 0.07 ms; Ib-600, 0.17 ± 0.11 ms), and *T*_2_ of the ^12^C, N-NDs (*T*_2_^mean^ = 3.2 ± 1.2 μs) was more than 11 times longer than those of the type-Ib NDs (Ib-100, 0.28 ± 0.10 μs; Ib-600, 0.21 ± 0.16 μs).

### Biological Applications

**Figure 4:**
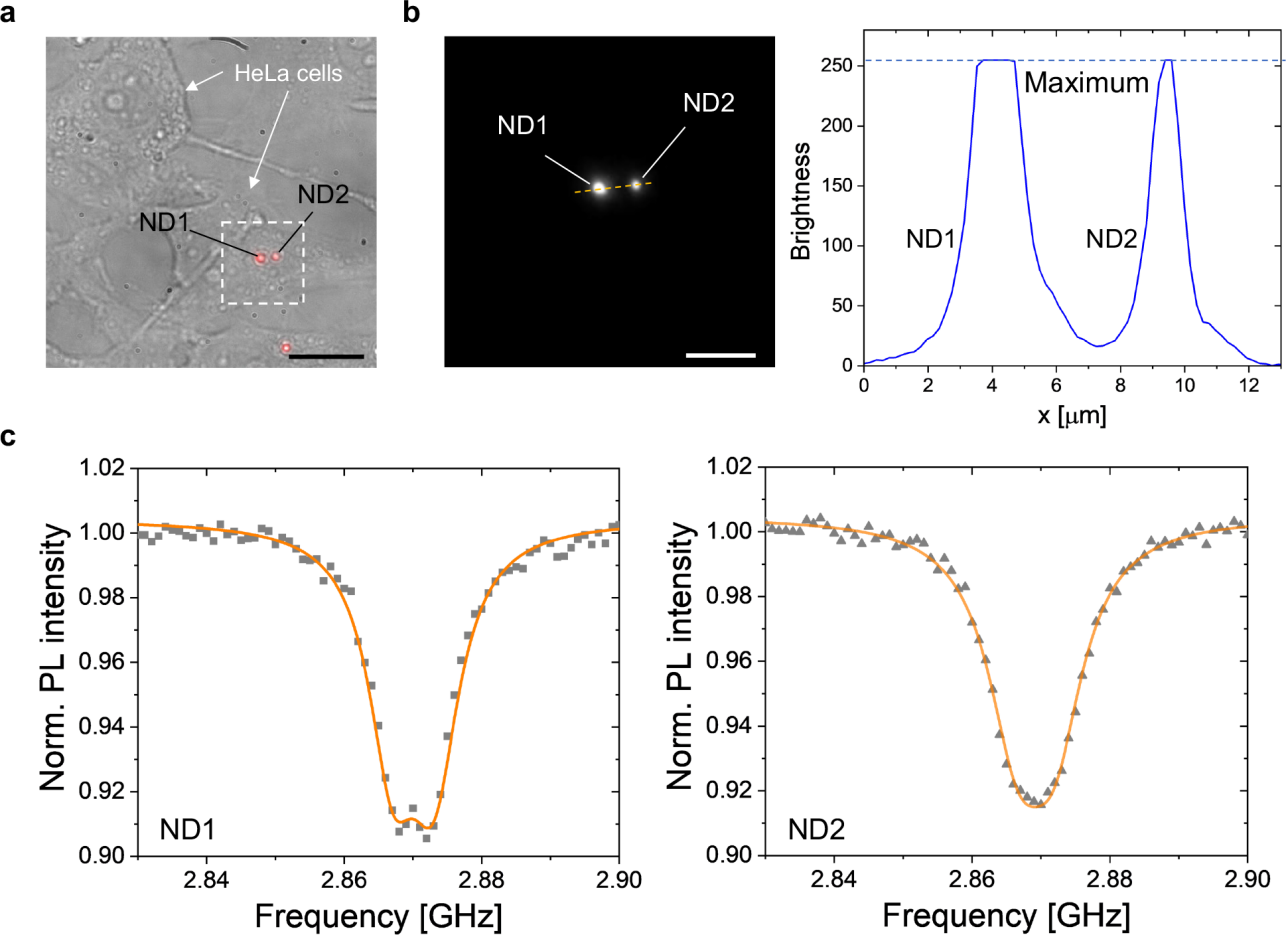
(a) Merged microscope image of gray bright-field and red fluorescence for HeLa cells uptaking ^12^C, N-NDs (scale bar: 25 μm). (b) Red fluorescence (ND1, ND2) in the white dotted box of Figure 5b (scale bar: 10 μm), and a cross-section along the yellow dotted line exhibiting the brightness of ND1 and ND2 (maximum value: 256). (c) In-situ ODMR spectra of ND1 and ND2 in the live cells, conducted in the absence of an external magnetic field.

The applicability of ^12^C, N-NDs to biological samples requires the introduction of NDs into live cells and NV spin detection. In this study, we fed prepared NDs to cultured HeLa cells to perform CW- and pulsed-ODMR experiments inside live cells. We fabricated a culture device by bonding a multiwell acrylic frame to coverslips with a notch-shaped antenna and cultured the HeLa cells in the wells (see Figure S10a). Subsequently, we introduced ^12^C, N-NDs into the cells via endocytosis (see Methods). Figure 4a shows a merged bright-field and red-fluorescence image of ND-labeled HeLa cells. The two NDs in Figure 4a (designated as ND1 and ND2) are sufficiently bright to distinguish in the presence of autofluorescence in the cells. Figure 4b indicates that the brightness of ND1 and ND2 on the yellow dotted lines in Figure 4a is saturated or near saturation, whereas the background fluorescence is nearly zero.

We measured and observed narrow and deep ODMR spectra of ND1 and ND2 inside the cells (Figure 4c). Fluorescence fluctuations owing to Brownian motion were observed during the CW-ODMR measurements (Figure S10c). We performed pulsed-ODMR measurements on ND2 under an external magnetic field in a similar manner to that described above. The Brownian motion significantly affected the pulsed-ODMR data when the microwave was applied because the random fluctuation of the ND orientation appeared to be dephasing during signal integration^40^ (see Supporting Information, Section S7). The profile of all-optical *T*_1_ relaxometry provided a value of 0.87 ms, being six times larger than the mean *T*_1_ value of type-Ib NDs (Figure S10d). In contrast, the relaxation profile of the π/2-spin–echo sequence exhibited a substantial shortening (135 ns) owing to Brownian motion, being 1 order of magnitude shorter than the mean *T*_2_ value determined above (Figure S10e). The difficulties associated with the Brownian motion of NDs during pulsed-ODMR measurements have been previously discussed;^41^ however, to date, this phenomenon has not been actively investigated owing to the very short *T*_2_ in type-Ib NDs. The NV spin coherence of the present ^12^C, N-NDs will enable to develop advanced pulsed-ODMR quantum-sensing protocols against the Brownian motion.

### Carr–Purcell–Meiboom–Gill (CPMG) and Thermal Echo (TE) Measurements

**Figure 5:**
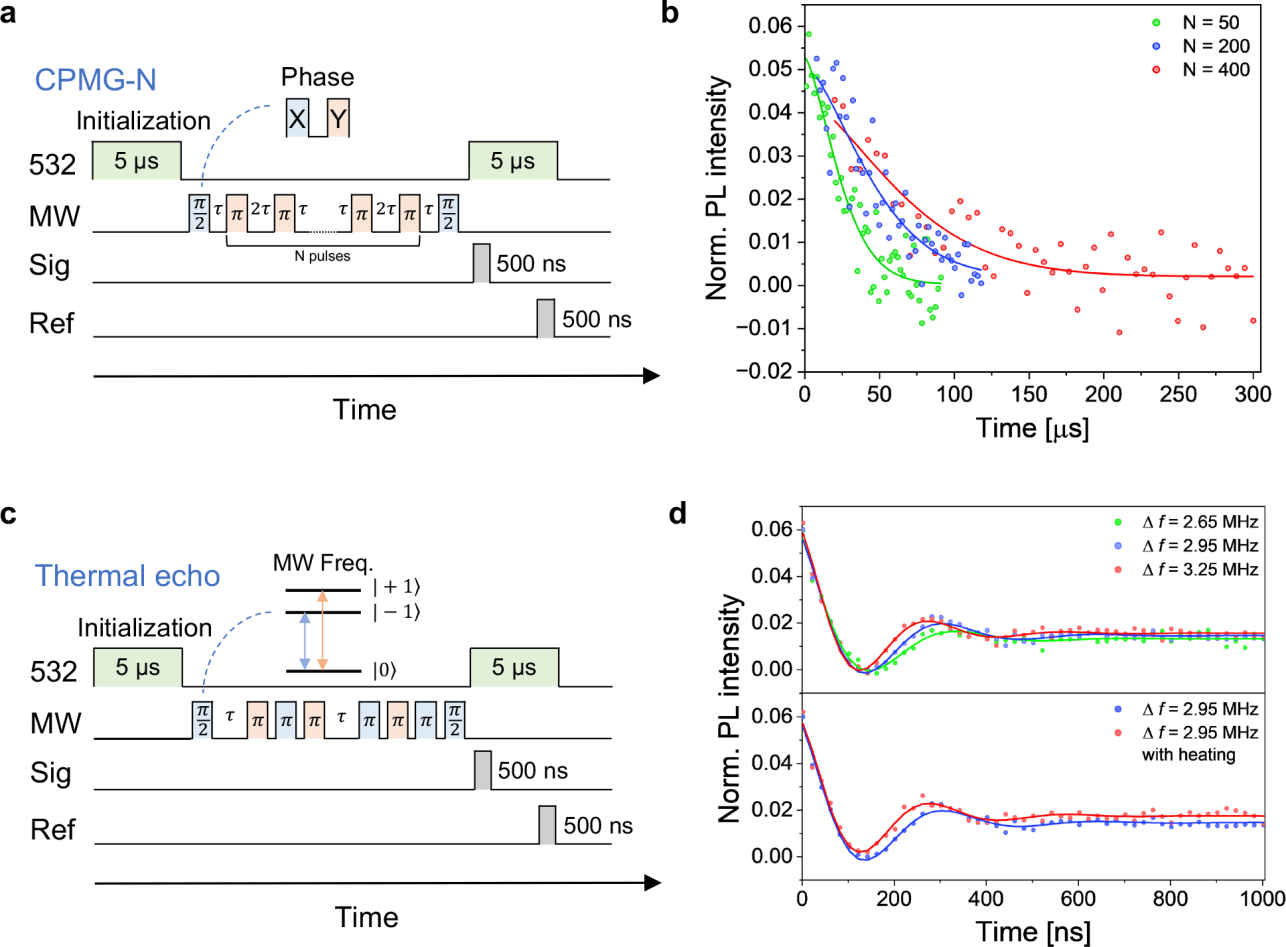
(a) Schematic sequences of N-pulse CPMG measurements. Red and blue indicate the *X* and *Y* phases of microwave pulses, respectively. (b) Representative profiles of N-pulse CPMG measurements for representative ^12^C, N-ND (green dots, *N* = 50; blue dots, *N* = 200; red dots, *N* = 400). The profiles were fitted using the stretched exponential decay, *A*_0_ exp((−τ/*T*_CPMG_)^*n*^) + *C*_0_ (*n* = 1.47 (green), 1.49 (blue), 1.50 (red)), where *A*_0_ and *C*_0_ are fitting parameters for each traces. (c) Schematic sequences of TE measurements. Red and blue indicate the microwave frequencies used for transitions |0⟩↔|+1⟩ and |0⟩↔|−1⟩ in the three-level diagram of NV centers, respectively. (d) (Top) TE measurements at room temperature (ca. 300 K) in external magnetic field illustrated with three different positive microwave frequency detuning values from zero-field splitting *D* (green dots, Δ*f* = 2.65 MHz; blue dots, Δ*f* = 2.95 MHz; red dots, Δ*f* = 3.25 MHz). (Bottom) TE measurements at room temperature (ca. 300 K) and temperature controlled to 308 K with detuning of Δ*f* = 2.95 MHz (blue dots, without stage heating; red dots, with stage heating). Both measurements were performed at the same detuning frequency to capture frequency changes according to temperature.

The NV spin properties of the ^12^C, N-NDs were further studied by advanced pulsed-ODMR experiments,^24^ including CPMG and TE measurements. The measurements involved the application of an external magnetic field to a representative ^12^C, N-ND. The eight resonances corresponding to the four NV quantization axes were observed (Figure S11a), followed by the pulsed measurements described in the Methods section. Using a representative ^12^C, N-ND exhibiting *T*_2_ = 3.3 μs (Figure S11c), we confirmed that *T*_2_ was extended by the CPMG sequences (Figure 5a) with more π-pulses (Figure 5b, *T*_CPMG_^*N*=50^ = 28.3 μs, *T*_CPMG_^*N*=200^ = 52.8 μs, *T*_CPMG_^*N*=400^ = 77.5 μs). Furthermore, we employed the enhanced *T*_2_ relaxation time of ^12^C, N-NDs (Figure 3f) in TE measurements. Figure 5c shows the sequences of TE measurements. To create oscillations of the TE signals, the applied microwave frequencies were detuned from zero-field splitting *D*. Figure 5d shows the TE results for detuning values (Δ*f* = *D* – Ω) of 2.65 MHz (green dots), 2.95 MHz (blue dots), and 3.25 MHz (red dots), where Ω denotes the microwave carrier frequency used for spin manipulation. Their observed oscillations in TE measurements were validated by fitting using function ∼ exp(−*t*/τ_*T*_TE__) cos(2π*ft*). The fitted oscillation frequencies were 2.66 ± 0.12 MHz, 2.97 ± 0.08 MHz, and 3.38 ± 0.10 MHz, respectively. Subsequently, TE measurements were conducted on the same ND with a frequency detuning of Δ*f* = 2.95 MHz at two different temperatures, namely, room temperature (ca. 300 K) and temperature controlled to 308 K. The room temperature of 300 K did not necessarily indicate the exact temperature of the ND (see Methods). Clearly, the oscillation frequency was changed from 2.97 ± 0.08 to 3.34 ± 0.11 MHz as the result of shift in *D* between the TE measurements (Figure 5d). The temperature change of 5.14 ± 1.35 K was determined by TE measurement, assuming a temperature dependency of d*D*/d*T* = −74 kHz/K. We inferred the corresponding temperature sensitivity as  considering observed *T*_TE_ = 147 ns and potentially reaching  if fully exploiting observed *T*_2_ = 3.32 μs^42,43^ (see Methods). This result demonstrates successful TE detection of shifts in *D* and precision determination by TE, utilizing oscillations at two different detuned frequencies.

## Discussion

This study demonstrates a remarkable improvement in the NV spin properties of bright NDs with [NV^–^] = 0.6–1.3 ppm. Microwave irradiation may result in heat generation and damage biological samples. Thus, the observed high contrast of the CW-ODMR spectra and up to a 20-fold reduction in microwave power (∼13 dB) are promising features for biological applications. For example, the reduction in microwave power can help mitigate microwave-induced toxicity, as a recent study reported that a difference of approximately 4 dB in microwave power substantially affected the cell viability of HeLa cells.^44^ The ^12^C, N-NDs exhibit long average spin-relaxation times of *T*_1_ = 0.68 ms and *T*_2_ = 3.2 μs, which are close to the bulk diamond limits and are longer than those of the conventional type-Ib NDs by factors of 6 and 11, respectively. This enhancement of the NV spin coherence is principally attributed to the spin-impurity control. We also confirm that *T*_2_ of the Ib-100 and Ib-600 NDs are close to the bulk-limited values calculated from their [NV^–^] = 300–540 ppm, which is estimated from their infrared absorption spectra (Figure S12b). The spin-impurity control is thus critical to improve the NV spin characteristics of the NDs with a size of ≳80 nm. The present *T*_1_ extension increases the relaxometric signal-to-noise ratio (SNR) in Gd^3+^ detection.^15^ Further, the ^12^C, N-NDs can improve the SNR by a factor of 6 compared with that of the type-Ib NDs, thus potentially lowering the detection limit for reactive oxygen species.^18^ Similarly, the 11-fold *T*_2_ extension increases the measurement sensitivity in AC magnetometry by a factor of 3,^45^ thus allowing the implementation of various *T*_2_-based quantum measurement protocols, such as advanced spin–echo quantum sequences in NDs.^31,46^

The proposed ^12^C, N-NDs pose some technical challenges, including: (i) the inhomogeneity in the NV characteristics should be further minimized, (ii) pulsed-ODMR protocols against Brownian motion must be established, and (iii) large-scale production of NDs is required. First, the inhomogeneity in the NV characteristics may arise from the spatial variations in the NV spins inside the original single crystalline diamonds because of several factors, such as different growth sectors, atomic concentrations, and dislocations.^47,48^ Further, surface spin noises can fluctuate the NV characteristics of each ND because inhomogeneous surface termination, e.g., different types and contents of dangling bonds, causes variations in the surface spin noise;^49,50^ these inhomogeneity factors may vary *T*_1_ and *T*_2_ (Figure 3e,f). In the present study, the variations in *T*_1_ and *T*_2_ observed in the case of the ^12^C, N-NDs are comparable with those for the Ib-100 and Ib-600 NDs. Therefore, these variations need to be further minimized to improve the measurement accuracy of quantum sensors employed in biomedical applications.^18^

Inhomogeneity in the NV spin environment compromises the precision of the microwave frequency used in TE measurements, crucial for detecting minor frequency changes. Additionally, a low  value results in broadening of the ODMR spectrum, highlighting the importance of a long  for precise selection of the microwave frequency in TE measurements. According to previous studies,  of the NV ensembles in high-NV-density type-Ib NDs was reported to be extremely short (<100 ns) and it could not be measured.^51^ Further, the measurement of pulsed ODMR, including Rabi oscillations and 3π/2-spin echo, becomes challenging when the ND size increases (Ib-100 and Ib-600; see Figures S9a–d and S13a) because of the increased inhomogeneity in the NV spin environment. In contrast, we observed clear Rabi oscillations and spin–echo inversion of the 3π/2-spin echo in the ^12^C, N-NDs (Figures S8j–l and S13a), indicating long  for the NV ensembles. Indeed, we observed Ramsey oscillations for a certain ^12^C, N-ND with  ns (Figure S13b,c). Although a statistical analysis was unfeasible because of the low success probability, the observed *T*_TE_ also exhibited a similar value of , indicating a short .

Second, precise detection of the resonance frequency in the ODMR spectrum is crucial for NDs experiencing Brownian motion. NDs exhibit Brownian motion inside the cells, and the rotation of the NV orientation relative to the microwave magnetic field fluctuates. During signal integration in the present measurement protocol, this effect modulates the spin signal, effectively shortening the microwave *T*_1_ and spin–echo decays.^40^ Additionally, there is a concern that the NDs could be aggregated in cells (Figure 4a,b). The optimal orientation relative to an external magnetic field can differ for each aggregated particle, significantly shortening both the *T*_1_ and *T*_2_ profiles when microwave spin manipulations are involved. All-optical *T*_1_ relaxometry is an exception as it relies solely on optical initialization. Therefore, it is imperative to develop an ND-specific ODMR measurement protocol that accounts for motion and random orientations.^52,53^

Finally, the large-scale production of ^12^C, N-NDs is required for further biochemical applications. Batch reactions for the surface functionalization of NDs require more than 10 mg of NDs to obtain higher yields.^10^ Currently, the number of single crystals with reduced spin impurities and high-density NV is insufficient because of the special production processes involving ^12^C methane and the absence of a mass production line. Furthermore, the milled NDs need to be centrifuged to separate the fractions for different sizes of NDs, which substantially reduces the final amount of NDs. The optimization of both the production process and the cost is important for achieving large-scale production.

## Study Limitations

This study demonstrates remarkably improved NV spin properties in bright NDs with [NV^–^] = 0.6–1.3 ppm. However, several limitations remain in this research. First, there is a challenge in accurately determining *T*_1_ and *T*_2_. Variations in these relaxation times are influenced by surface spin noises and inhomogeneities within the original crystal, such as surface termination, varying growth sectors, atomic concentrations, and dislocations. As  is basically shorter than *T*_2_ by these decoherence factors, we similarly hypothesize that *T*_TE_ is shorter than *T*_2_ due to these factors. Second, the limited production of ^12^C, N-NDs restricts comprehensive analyses, including X-ray photoelectron spectroscopy, DLS, and transmission electron microscopy. These analyses are crucial for further detailed material characterization of ^12^C, N-NDs. Third, the random orientation and motion of the NV quantization axis present challenges for performing pulsed ODMR. Fourth, the TE measurements in this study were conducted under stationary conditions for a representative ^12^C, N-ND. For example, the calibration for the TE-based temperature measurements requires more systematic experiments including the determination of temperature dependency of *D* for NDs and precise temperature control of the samples. To fully exploit the potential applications of ^12^C, N-NDs, the challenging experimental approaches, such as agile manipulation of microwave polarization and magnetic field direction are essential.

## Conclusions

In conclusion, we demonstrated the development of bright spin-impurity controlled NDs containing  = 0.6–1.3 ppm with spin impurities of [^12^C] = 99.99% and [N] = 30–60 ppm. The NDs exhibited a 50–700 nm size range and were readily used for the fluorescent labeling of cultured cells. We demonstrated a remarkable improvement of the NV spin quality as compared to that of the conventional type-Ib NDs including narrow and deep CW-ODMR spectra and the extended average spin-relaxation times of *T*_1_ = 0.68 ms and *T*_2_ = 3.2 μs (1.6 ms and 5.4 μs in maximum), approaching the bulk limit. Furthermore, TE measurements with ^12^C, N-NDs showed a temperature sensitivity of , a level of sensitivity not achieved with bare type-Ib NDs. We observed that these NDs used 20 times less microwave power to reach a 3% ODMR contrast than their type-Ib counterparts. Using these ^12^C, N-NDs, we performed ODMR measurements (CW-ODMR, *T*_1_ and *T*_2_) inside the cells. These results successfully demonstrate the pertinence of quantum-grade NV spin properties for quantum sensing in potential biological applications.

## Methods

### ND Preparation

^12^C-isotope-enriched single-crystalline bulk diamonds with controlled nitrogen concentrations were synthesized using the HPHT method as described previously,^54^ with minor modifications for single-crystal growth. The ^12^C enrichment was 99.99% and the nitrogen concentration was 30–60 ppm, as confirmed via secondary ion mass spectroscopy (SIMS) after HPHT growth. NV centers were generated in these bulk diamonds using electron beam irradiation (3 MeV, 1 × 10^18^ cm^–18^) under ambient conditions, followed by vacuum annealing at 900 °C for 1 h.^55^ These bulk diamonds were pulverized, followed by suspension in water to obtain ^12^C, N-NDs. Note that ^12^C N-ND surface was nontreated after pulverization because of the limited-production. Type-Ib NDs with mean sizes of 100 and 600 nm were purchased from Adámas Nanotech. (NDNV100 nmHi, NDNV600 nmHi).

### ND Coating on the Coverslips and AFM

To determine the NV concentration of ^12^C, N-NDs, coverslips with engraved island grids on one side was used. A small droplet of the ND suspension was drop-casted onto a coverslip, enabling the utilization of the same NDs for brightness confirmations and AFM measurements. The topographies of the spin-coated samples were determined using AFM in the tapping mode (SPA400, Hitachi High-Tech Corporation) after the ODMR measurements. The height of the ND topography was regarded as the ND size to avoid the tip convolution effect.^56−58^ The images were collected at a scan rate of 0.1–0.5 Hz.

### Optical and ODMR Measurements

The optical properties and ODMR of the NDs were measured using a lab-built confocal fluorescence microscope with a microwave excitation system based on previous studies.^34,38,59^ A 532 nm laser was used to excite the NDs with an intensity in the 5–10 kW cm^–2^ range, which corresponds to an optical saturation parameter of *s* = 0.05–0.10.^38,60^ For the excitation and the fluorescence collection, a 50× dry objective with a numerical aperture of 0.7 mounted on a piezo actuator (Piezosystemjena, MIPOS 100) for fine *z*-axis adjustment was used. A voice-coil-driven fast-steering mirror (Optics In Motion, OIM101) was used for fast xy-scanning of the laser. NV fluorescence was filtered using a dichroic beam splitter (Semrock, FF560-FDi01) and a long-pass filter (Semrock, BLP01-635R-25) to remove residual green laser scattering. The fluorescence was coupled to an optical fiber (Thorlabs, 1550HP) and detected using a single-photon counting module (Excelitas, SPCM-AQRH-14), and its spectra were measured using a spectrometer equipped with a charge-coupled device camera (Princeton Instruments, PIX256OE-SF-Q-F-A). The output from the photon-counting module was fed into a board system (National Instruments USB-6343 BNC). The laser scanning was controlled using a lab-built program using GPScan^61^ in part. In the ODMR measurements for both the continuous-wave (CW) and pulsed modes, microwaves were generated using a signal generator (Rohde and Schwarz, SMB100A) and sent to radiofrequency (RF) switches (Mini-circuit, ZYSWA-2–50DRS and General Microwave, F9160) triggered by a bit-pattern generator (SpinCore, PBESR–PRO-300). The signal was then amplified using a 45 dB amplifier (Mini-circuit, ZHL-16W-43+). In the CW mode, microwave excitation was gated using the RF switches to suppress noise (200 μs for microwaves on and off). In the pulsed mode, external magnetic fields of 5–15 G were applied along the NV quantization axis using a small neodymium magnet. The magnet was mounted on a multiaxis manual stage to lift the degeneracy of the magnetic sublevels, thereby enabling sufficient ODMR contrast for the Rabi-nutations in the subsequent pulsed measurements. An external magnetic field was applied to split the CW-ODMR into two peaks, facilitating the detection of Rabi signals necessary for determining the π-pulse in subsequent pulsed measurements. The pulse operation of the excitation laser was implemented using an acousto-optic modulator (AOM, G&H, 3200-121), and NV spins were optically initialized using a 3 μs pulse width. All pulse sequences are presented in Figure S4b–d. To determine the duration of π pulse for the NV spins, Rabi measurements were performed, and the profile was fitted with a sine-damping function. *T*_2_ was measured using spin–echo sequences, and π/2−π–π/2 (π/2-spin echo) and π/2−π–3π/2 (3π/2-spin echo) sequences were measured to cancel common-mode noise.^62^*T*_1_ was measured using spin-polarization relaxometry sequences. We measured all-optical *T*_1_ and microwave *T*_1_ relaxometry sequences, separately and canceled the common-mode noise by subtraction.^39^

### CW-ODMR Spectral Analysis

The observed ODMR spectra were fitted using a double-Lorentzian function (*y* (*x*)) composed of two Lorentzian functions, *L*_1,2_ (*x*): *y* (*x*) = *y*_0_ + *L*_1_ (*x*) + *L*_2_ (*x*), and , where, *y*_0_, *A*_1,2_, *w*_1,2_, and *x*_1,2_ are the offset, peak area, line width, and peak position frequency of *L*_1,2_ (*x*), respectively. The following boundary conditions were applied to the fitting: 2.862 ≤ *x*_1_ ≤ 2.870 and 2.870 ≤ *x*_2_ ≤ 2.878. The “Norm. PL intensity” in Figure 2e were determined by taking the mean of the two peaks [*y*(*x*_1_) + *y*(*x*_2_)]/2. In this study, the spectral parameters for the peak splitting *E* and zero-field splitting *D* were determined using the fitting parameters *E* = *x*_2_ – *x*_1_ and *D* = (*x*_1_ + *x*_2_)/2, respectively (Figure 2f,g). *E* is the strain-induced parameter.^63−65^ Further details of the fitting procedure are provided in the Supporting Information, Section S4.

### Analysis Method for the *T*_1_ and *T*_2_

The raw *T*_1_ relaxation profiles showed large amount of noise, which destabilized the subsequent fitting processes. These noises were numerically filtered by taking a moving average over nine data points (out of a total of 50 points), and the filtered profiles measured with the all-optical *T*_1_ and microwave *T*_1_ sequences were subtracted (see Figure S8a–i). The subtracted *T*_1_ profiles were fitted using a two-phase exponential decay, *C* = *C*_0_ + *A*_1_ exp(−(τ – *t*_0_)/*t*_1_) + *A*_2_ exp(−(τ – *t*_0_)/*t*_2_), where *C*_0_ is the common offset and *A*_1,2_, and *t*_1,2_ are amplitudes, and time constants, respectively. Out of the two decay times, the longer one was used as *T*_1_ (*T*_1_ = max(*t*_1_, *t*_2_)) as previously described.^66−70^ Similarly, the π/2- and 3π/2-spin–echo profiles were filtered by moving the average over five data points (out of a total of 50 points) before their subtraction. For the ^12^C, N-NDs, the subtracted profiles were fitted using the stretched exponential decay exp((−2τ/*T*_2_)^1.5^) to determine *T*_2_(28) (see Figure S6a–c). For the *T*_2_ determination of the type-Ib NDs, this fitting is only applied to the π/2-spin echo profiles because 3π/2-spin echo sequence did not provide spin–echo amplitudes owing to the short  (see Supporting Information, Section S6).

### Statistical Analysis

Mean values and standard deviations (1σ) are displayed in the statistical plots. Statistical significance among three independent samples (Ib-100, Ib-600, ^12^C, N-NDs) was analyzed using the Kruskal–Wallis test with Dunn’s test for multiple comparisons. Significant differences were identified where the p-value was less than 0.05 (*p* < 0.05). All analyses were conducted using Origin.

### Thermal Echo and CPMG Measurements

A series of experiments related to thermal echo and CPMG measurements were conducted using a home-built confocal microscopy at Institute for Quantum Life Science (iQLS), QST, Japan.^71^ The breadboard was placed inside Olympus IX-73 to guide the laser into the objective lens through a dichroic mirror. The detector side could be switched to an EM-CCD camera (iXonUltra) or a pinhole with an APD detector (SPCM-AQRH-14-FC-ND) or a color CCD camera (E3CMOS) through the adjuster placed on the lower deck of IX-73. The incubator was placed inside the piezo stage (P-545.3C8S) to control the temperature with a thermocoupled-heater to change the temperature of the system. Microwaves were delivered from the SG (Anritsu MG3700A and N5182A) combined with an amplifier (ZHL-50W-63+), and microwaves were delivered to the NV center through a 20 μm diameter copper wire with a sputtered Ti/Cu/Au electrode on a cover slide. The pulse sequence was controlled by DTG5274 and the MW pulse was truncated by a switch (Mini circuit ZASWA-2-50DRA+). A high-power laser (Verdi G5) was pulsed through an AOM (Gooch Housego, Model: 3250–220) with an RF driver (3910-XX). In the TE measurements using ^12^C, N-NDs, an external magnetic field was applied along the direction close to the [111] NV quantization axis and the eight resonances corresponding to the four NV quantization axes were obtained. The applied microwave frequencies were detuned, as previously reported.^71^ The temperature was set to 308 K from room temperature and the system reached thermal equilibrium within 30 min. Note that the sample temperature could not be precisely controlled by a temperature controller at a stage holder as it was positioned away from the sample. For estimating the temperature sensitivity and assuming d*D*/d*T* = −74 kHz/K and reported parameter *S*(300 K) ∼ 0.02 accounting for finite photon count rate and electron spin resonance contrast, we used *T*_TE_ = 147 ns for the sensitivity equation available for bulk diamonds.^42,43^ The potentially reachable sensitivity was calculated considering *T*_2_ = 3.32 μs (Figure S11c).

### ND Labeling of HeLa Cells

HeLa cells were cultured in a cell culture medium (DMEM containing 4500 mg/mL glucose, 10% FBS, 100 U mL^–1^ penicillin/streptomycin, and phenol red) in a well that was fabricated on a coverslip, with a notch-shaped antenna on the other side (see Figure S10a). The glass surface of the device (well side) was coated with collagen to improve cell adhesion. ND labeling of cells was performed using the method described previously.^72,73^ A suspension of ^12^C, N-NDs was added to the culture medium, and the cells were incubated at 37 °C and 5% CO_2_ for 24 h. The cells were then washed gently three times with phosphate-buffered saline and immersed in a culture medium without phenol red to perform the ODMR measurements.

### FTIR Measurements

The nitrogen concentration in the type-Ib NDs was estimated by measuring 150-μm-sized type-Ib diamond microcrystals (Adámas Nanotech., MDNV150umHi) from the same product line using the Fourier transform infrared (FTIR) spectroscopy, as previously reported.^74,75^ A Jasco FTIR6200-IRT7000 micro Fourier-transform spectrometer was used with a KBr/Ge beam splitter, a ceramic light source, and MCT detector. The microcrystals were placed on a copper mesh with pore sizes of 85 μm, and near-infrared (NIR) light was focused with a spot size of 50–100 μm through Cassegrain optics in the transmission mode. The sample chamber was purged with nitrogen gas to minimize atmospheric background in the spectra. The spectral data were accumulated 512 times with a spectral resolution of 4 cm^–1^. From the obtained spectrum, the nitrogen concentration [N] (ppm) was determined using the following relationship: () × 5.5 × 25, where μ is absorption intensity for the given wavenumber.^76^

## Supporting Information Available

Determination of the NV concentration in NDs with size–brightness correlation; ODMR experimental setup; loss/gain analysis of microwave excitation system including antenna characterization; ODMR spectral analysis using a double-Lorentzian function; Raman measurements; fitting procedure of the relaxation curves; cell culture protocol and other experimental results; series of experiments related to thermal echo measurements; determination of nitrogen concentration for type-Ib NDs from FTIR spectrum; Rabi oscillation comparison, and Ramsey experiments (PDF)
