# Supplementary material for: Bright Quantum-Grade Fluorescent Nanodiamonds
Source: ACS Nano. 2024 Dec 16;18(52):35202–13. doi: 10.1021/acsnano.4c03424 (PMC11697348; doi:10.1021/acsnano.4c03424)
Supplement: Supplementary file 1 — nn4c03424_si_001.pdf [file nn4c03424_si_001.pdf]

Supporting Information: Bright quantum-grade fluorescent nanodiamonds

Keisuke Oshimi,[1,*] Hitoshi Ishiwata,[2,*] Hiromu Nakashima,[1] Sara Mandić,[1] Hina Kobayashi,[1] Minori Teramoto,[3] Hirokazu Tsuji,[3] Yoshiki Nishibayashi,[3] Yutaka Shikano,[4,5,6] Toshu An,[7] and Masazumi Fujiwara[1,†]

[1] *Department of Chemistry, Graduate School of Life, Environmental, Natural Science and Technology, Okayama University, Okayama 700-8530, Japan*
[2] *The National Institutes for Quantum Science and Technology (QST), Institute for Quantum Life Science (iQLS), Chiba 263-8555, Japan.*
[3] *Advanced Materials Laboratory, Sumitomo Electric Industries, Ltd., Hyogo 664-0016, Japan*
[4] *Institute of Systems and Information Engineering, University of Tsukuba, Tsukuba, Ibaraki 305-8573, Japan*
[5] *Center for Artificial Intelligence Research (C-AIR), University of Tsukuba, Tsukuba, Ibaraki 305-8577, Japan*
[6] *Institute for Quantum Studies, Chapman University, Orange, CA 92866, USA*
[7] *School of Materials Science, Japan Advanced Institute of Science and Technology, Nomi, Ishikawa 923-1292, Japan*

S1. Determination of the NV concentration in NDs with size–brightness correlation

Figures S1**a**–**d** show AFM topology and confocal images of the type-Ib NDs (Ib-100, Ib-600). These AFM topography images, including Figs. 1**d, e** for ^{12}C, N-NDs, have a common artifact: convolution caused by interaction between sample and AFM tip. Figure S1**e** shows a schematic of this convolution effect in the AFM measurements of NDs. The experimentally determined width of the NDs (W_{exp}) in the xy-imaging plane can be written as [1]:

$$\begin{aligned} \frac{1}{2}W_{exp} &= r_{tip} + \Delta + \frac{1}{2}W_0 \\ \Delta &= (H - r_{tip})\tan\theta \end{aligned} \tag{S1}$$

where W_0, H, r_{tip}, and θ are ND width in the xy-imaging plane, ND's height from the xy plane (glass surface), tip radius, and tip angle, respectively. The term of $r_{tip} + \Delta$ indicates the tip convolution. From this analysis, we calculated W_{exp}/W_0 to show how much convolution effect exists in the present three types of NDs (Ib-100, Ib-600, ^{12}C, N-NDs). As shown in Fig. S1**f**, the results indicate that the convolution effect is comparable to each other among these samples. The platelet morphology of NDs was confirmed by scanning electron microscopy (SEM) images (Fig. S2), and we adopted the mean values of the widths along the x and y axes ($L_{1,2}$) as W_{exp} for simplicity.

After determining W_0, we calculated [NV$^-$] as follows. We first assumed that the fluorescence intensity is proportional to the number of NVs as the optical excitation intensity is far below the saturation ($s \approx 0.05$). As the number of NVs is proportional to the ND volume, the fluorescence intensity can be written as

$$I \propto [\mathrm{NV}^-]V, \tag{S2}$$

where I and V are the fluorescence photon-count rate and the volume of NDs, respectively. By taking a ratio of the fluorescence intensity for the two types of the NDs, [NV$^-$] can be determined as follows:

$$[\mathrm{NV}^-]_j = [\mathrm{NV}^-]_i \frac{I_j}{I_i}\frac{V_i}{V_j}, \tag{S3}$$

where the subscripts i, j indicate the type of the NDs. By introducing aspect ratio ($A = W_0/H$), V can be expressed as

$$V = kH^3 g(A) \tag{S4}$$

where k and $g(A)$ denote morphology dependent factor and A-dependent conversion factor, respectively (e.g, $k = \pi/6$, $g(A) = A^3$ for sphere, $k = 1$, $g(A) = 1$ for cube, $k = \pi/4$, $g(A) = A^2$ for cylinder, as shown in Fig. S3**a**).

---

* These authors contributed equally to this work.

† Corresponding author: masazumi@okayama-u.ac.jp

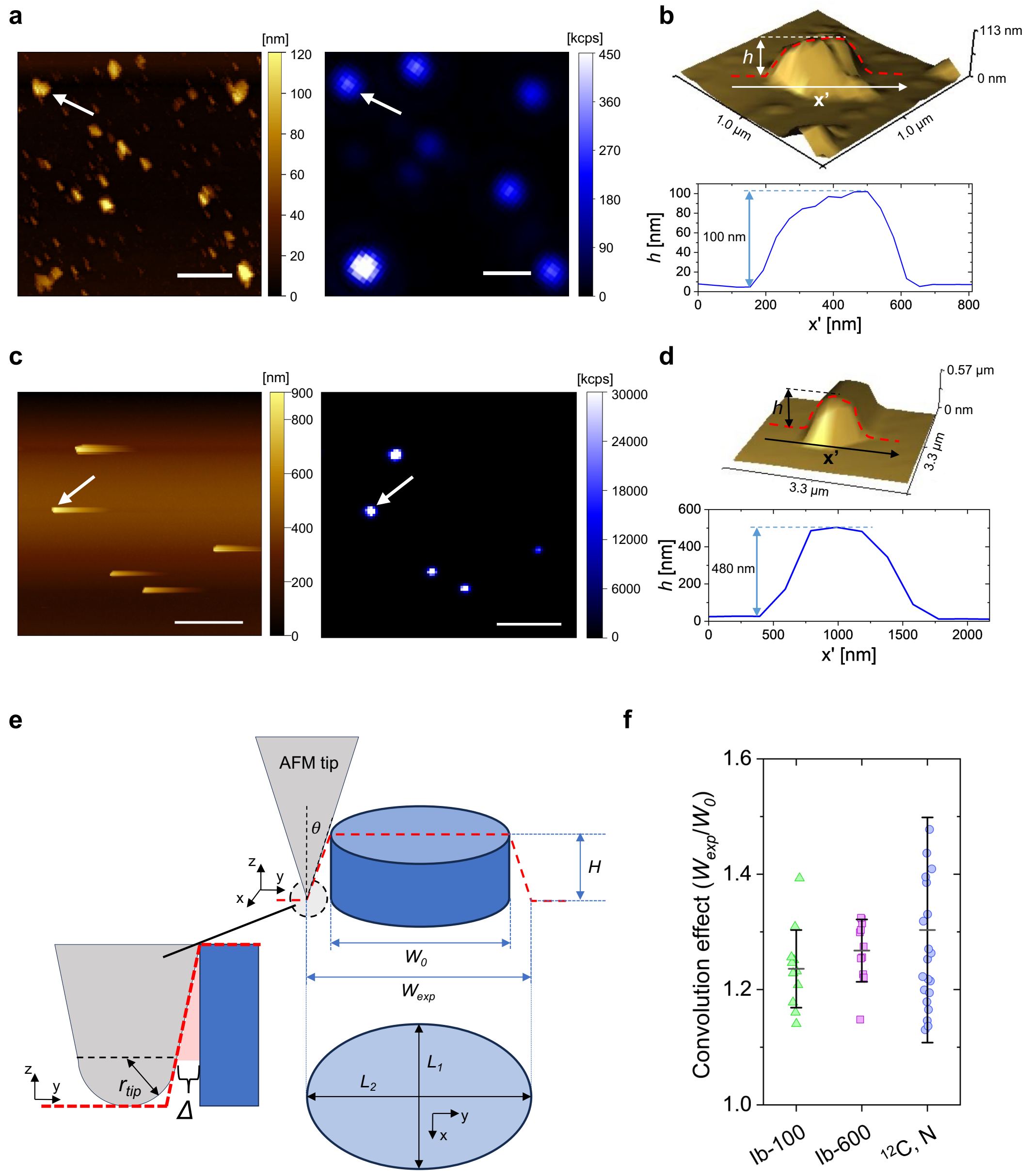

FIG. S1. (a) AFM topography (left) and the corresponding confocal fluorescence (right) images of Ib-100. Scale bar: 1 μm. (b) Three-dimensional visualization of the topography of the ND indicated by the white arrow in Fig. S1**a** (top) with a cross-section along x' axis (bottom). (c) AFM topography (left) and the corresponding confocal fluorescence (right) images of Ib-600. Scale bar: 10 μm. (d) Three-dimensional visualization of the topography of the ND indicated by the white arrow in Fig. S1**c** (top) with a cross-section along x' axis (bottom). (e) Schematic of the AFM measurements with the convolution effect. W_{exp}, W_0, H, r_{tip}, and θ are the experimental, true length in the xy-imaging plane of the measured sample, height of the sample from the xy-imaging plane, AFM tip radius, and tip angle, respectively. L_1 and L_2 are the ND width in the x and y axes, respectively. $r_{tip} = 10\,\text{nm}$, $\theta = 20°$. Red dotted line: the convolution effect of the AFM tip. (f) Statistical plots of the convolution effect (W_{exp}/W_0) for Ib-100, Ib-600 and ^{12}C, N-NDs. Mean and standard deviation (1σ) are indicated in the statistical plots.

We then estimated the NV concentration of the ^{12}C, N-NDs. In Fig. 1**g**, the fluorescence photon-count rate of Ib-600 NDs was approximately 150 times more than that of Ib-100 ($I_{\text{Ib600}}/I_{\text{Ib100}} \approx 150$). Provided both of the ND morphology as $A = 1$ such as cubic, Ib-600 NDs were found to be approximately 130 times larger in volume than Ib-100 NDs ($V_{\text{Ib600}}/V_{\text{Ib100}} \approx 130$). Through Eq. S3 and Eq. S4, these parameters give $[\text{NV}^-]_{\text{Ib600}} \approx 1.2[\text{NV}^-]_{\text{Ib100}}$, comparable to the ratio determined from their specification sheet ($[\text{NV}^-]_{\text{Ib100}} = 3\,\text{ppm}$, $[\text{NV}^-]_{\text{Ib600}} = 3.5\,\text{ppm}$). Similarly, assuming the premise of ^{12}C, N-NDs having $A = 1$ type morphology, we estimated $[\text{NV}^-]_{^{12}\text{C},N}$ to be

$0.26[\mathrm{NV}^-]_{\mathrm{Ib100}} \approx 0.6$ ppm or $0.20[\mathrm{NV}^-]_{\mathrm{Ib100}} \approx 0.8$ ppm by taking a reference to Ib-100 or Ib-600 NDs, respectively.

We also estimated $[\mathrm{NV}^-]_{^{12}\mathrm{C},N}$ for a $A \neq 1$ morphology. For the subsequent volume calculation using W_0, by replacing the elliptical contour of the platelet shape with circle, we assumed each particle to have a cylinder shape with its dimensions estimated to yield the same volume ($k = 1$, $g(A) = A^2$ in Eq. S4). Figure S3**b** shows statistical plots of A by analyzing the AFM data of the NDs. From this data, we obtained A^{mean} for Ib-100, Ib-600 and ^{12}C, N-NDs to be 3.9 ± 1.2, 2.9 ± 0.8 and 3.3 ± 1.6, respectively. These values are comparable to the previous study about 140 nm NDs, where the NDs have $A \approx 3$ [2]. Note that we analysed only one length of NDs (either of L_1 and L_2) of Ib-600 using the tailed AFM topography image caused by the measurement limitation of AFM tapping mode (Fig. S1**c**), so that it is possible that the calculated aspect ratio of Ib-600 can become slightly smaller value than that of Ib-100. Figures S3**c**–**e** show volume-brightness correlation plots of the type-Ib NDs and ^{12}C, N-NDs for $A = 1$ and $A \neq 1$. The scatter plots were entirely shifted to the lager side for all of the ND types, when the volume estimation method was switched from $A = 1$ to $A \neq 1$. Using this platelet shape with $A \neq 1$, we obtained $0.44[\mathrm{NV}^-]_{\mathrm{Ib100}} \approx 1.3$ ppm or $0.21[\mathrm{NV}^-]_{\mathrm{Ib600}} \approx 0.6$ ppm by taking a reference to Ib-100 or Ib-600 NDs, respectively. Therefore, we determined $[\mathrm{NV}^-]$ of the present ^{12}C, N-NDs was 0.6–1.3 ppm.

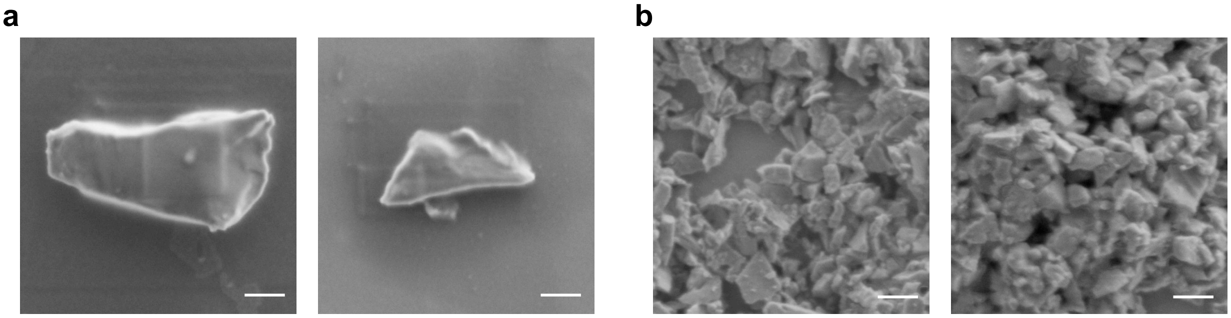

FIG. S2. SEM images with the acceleration voltage 1.00 kV for (a) ^{12}C, N-NDs and (b) Ib-100 NDs. Scale bar: 200 nm

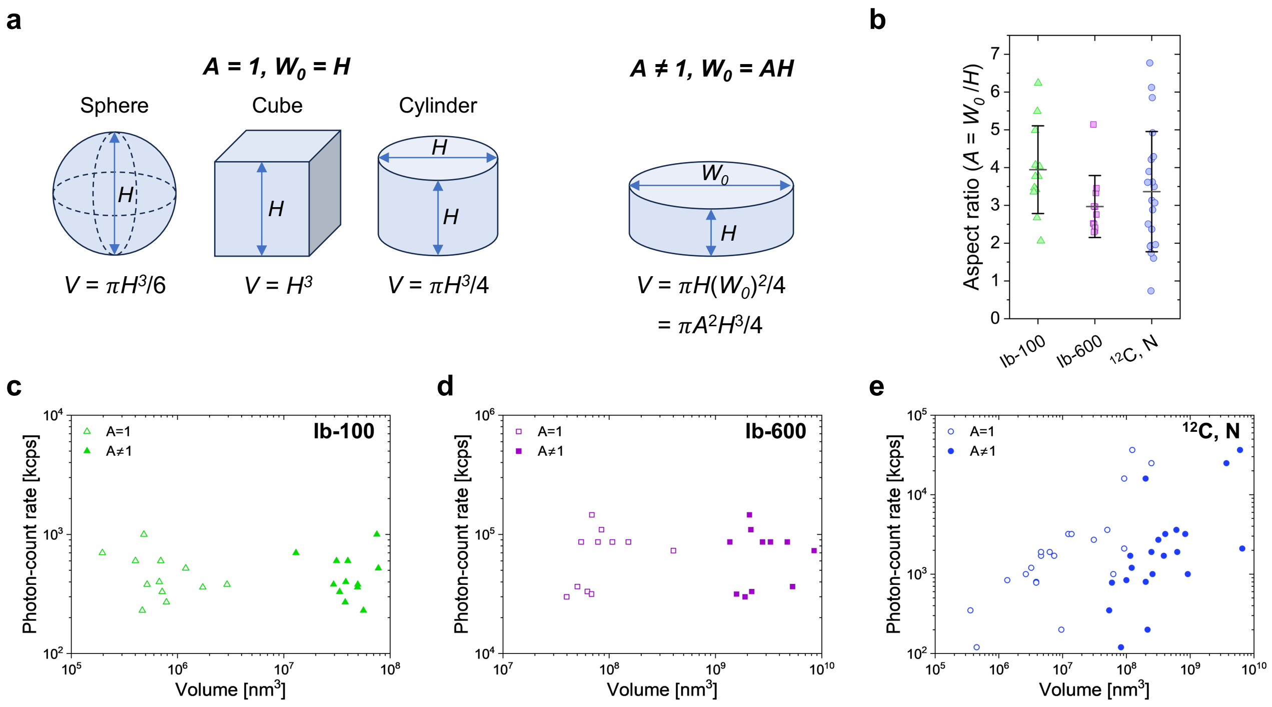

FIG. S3. (a) Schematic images of the typical ND morphology with their parameters defining the shapes. A, V, H, and W_0 indicate aspect ratio, volume, width, and height, respectively. (b) Statistical plots of the aspect ratio for Ib-100, Ib-600 and ^{12}C, N-NDs. Mean and standard deviation (1σ) are indicated in the statistical plots. Plots of the ND photon counts against the ND volume in the case of the cylinder with $A = 1$ and $A \neq 1$ for (c) Ib-100, (d) Ib-600, (e) ^{12}C, N-NDs.

S2. ODMR experimental setup

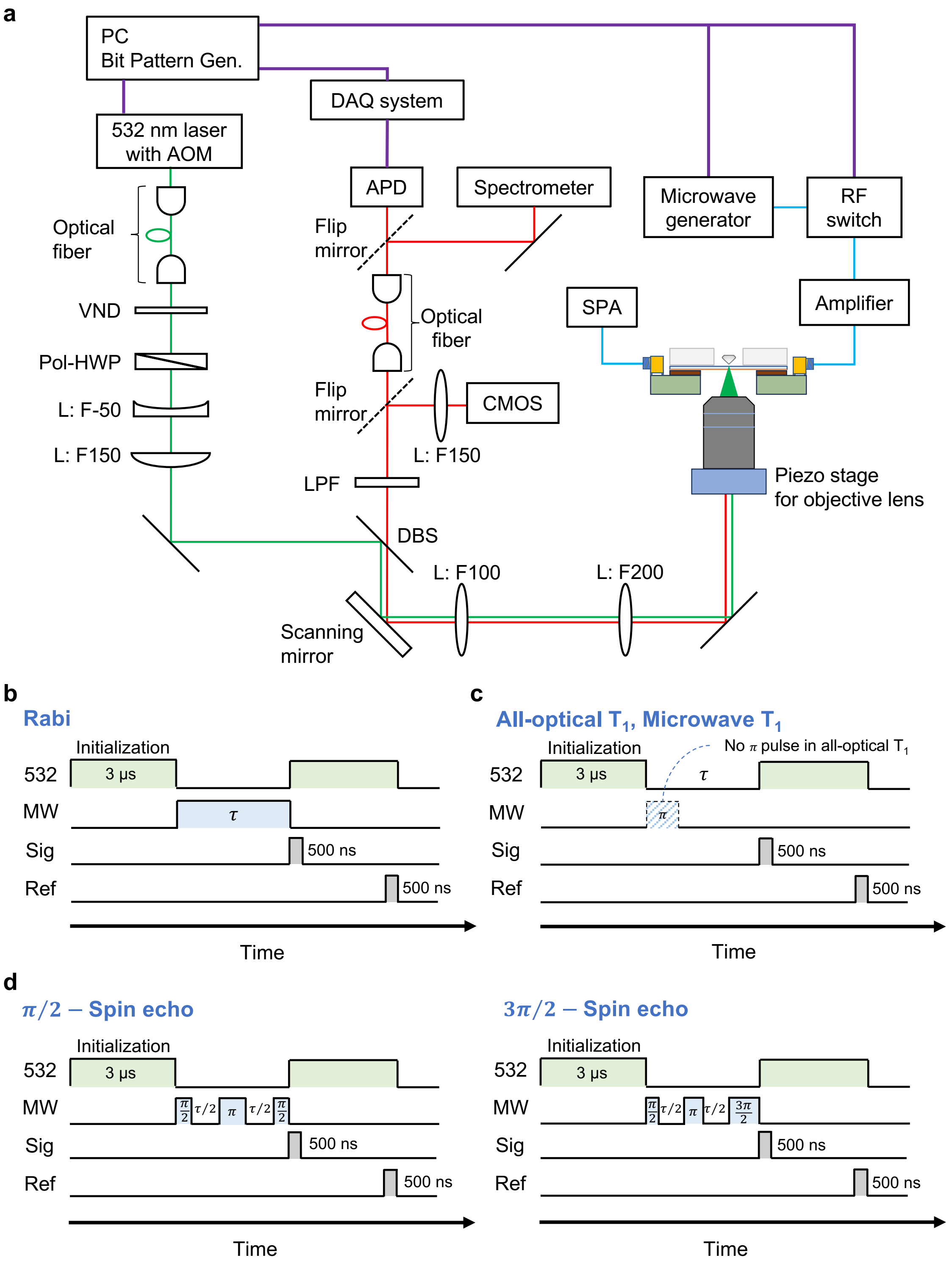

FIG. S4. (a) Schematic of the ODMR setup comprising confocal fluorescence microscope and microwave excitation system. AOM: acousto-optic modulator. VND: variable neutral density filter. Pol-HWP: polarizer and half-wave plate. L: lens. DBS: dichroic beam splitter. LPF: long pass filter. CMOS: complementary metal oxide semiconductor. APD: avalanche photodiode. SPA: spectrum analyzer. RF: radio frequency. Schematic pulse sequences for (b) Rabi, (c) all-optical T_1, microwave T_1, and (d) $\pi/2$, $3\pi/2$-spin echo measurements.

S3. Loss/gain analysis of microwave excitation system including antenna characterization

Figures S5**a, b** show the geometrical structure of a notch-shaped antenna on a coverslip and the experimentally measured S-parameters, respectively. Table S1 summarizes the losses and gains of the individual components of the microwave system in the ODMR setup. The microwave losses in SMA cables and RF switches were obtained from their specification sheets, and we assumed a minimum gain of +40 dB for the amplifier, considering the high-impedance matching between the cables and the antenna. The loss of the antenna on the PCB at the input (L_R) and output ports (L_T) was calculated from the observed S_{11} and S_{21} values using the following equation [3]:

$$L_R = 10\log_{10}(1 - 10^{\frac{S_{11}}{10}}), \tag{S5}$$

$$L_T = \frac{S_{21}}{2}. \tag{S6}$$

The applied microwave power was calculated from the corresponding S-parameter values (Fig. S5**b–d**), and they were converted from dBm to mW.

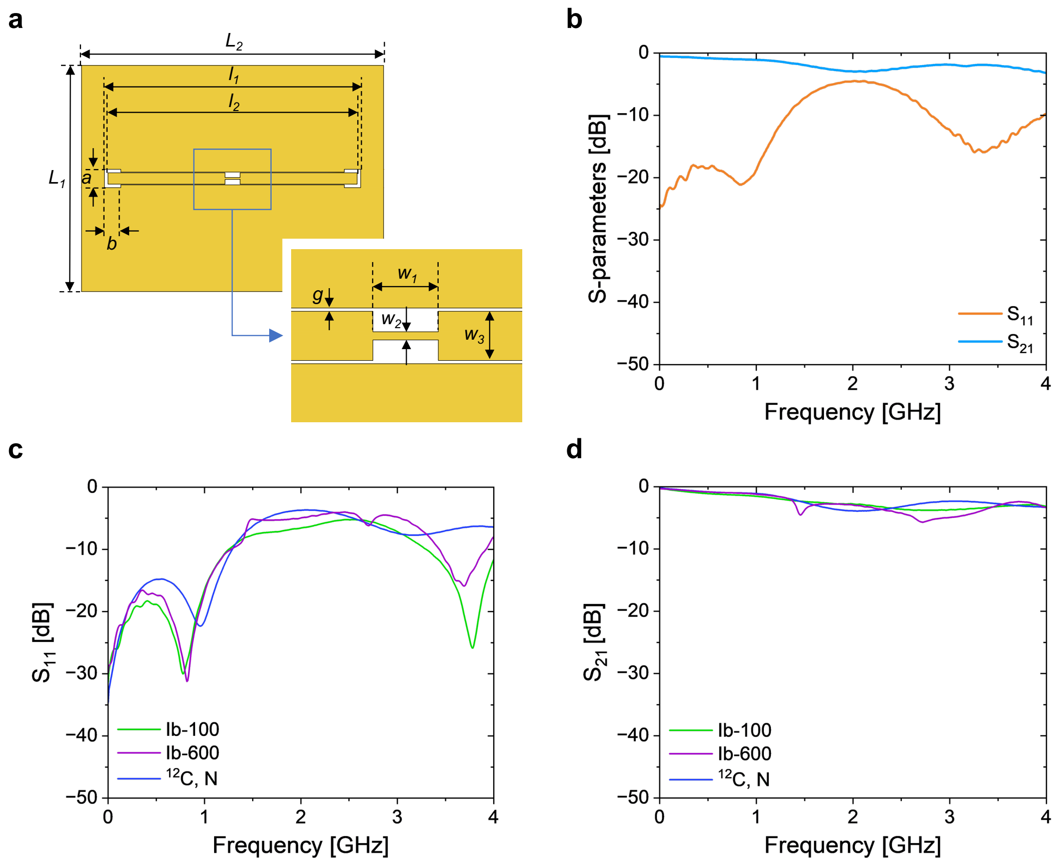

FIG. S5. (a) Geometrical structure of the notch-shaped antenna patterned on the coverslip with dimensions as follows: $L_1 =$ 30 mm, $L_2 = 40$ mm, $l_1 = 33.94$ mm, $l_2 = 33$ mm, $a = 2.44$ mm, $b = 2.17$ mm, $g = 0.10$ mm,$w_1 = 2.0$ mm, $w_2 = 250$ µm, and $w_3 = 1.5$ mm. (b) Experimentally measured S_{11} and S_{21} spectrum in the experiment of Fig. 2**c** (c) Experimentally measured S_{11} and (d) S_{21} spectrum of the antenna used for the experiments of type-Ib NDs and ^{12}C, N-NDs presented in Figs. 2**d**–**g**.

TABLE S1. List for microwave input power, loss and gain of the microwave components

Components	Loss or Gain [dB]	Power [dBm]	Notes
Microwave generator		−16.0	The experiment of the Fig. 2**c** (bottom)
RF switches	−3.5	−19.5	Total insertion loss
Microwave amplifier	+40.0	20.5	Amplifier gain
Microwave cables	−5.0	15.5	Total insertion loss
Antenna reflection loss	−0.386	15.1	$S_{11} = -11.0$
Antenna insertion loss	−0.955	14.2	$S_{21} = -6.32$
Total in the detection area		14.2 [dBm]	

The magnetic field of the microwaves ($|\boldsymbol{B}|$) on the antenna in Fig. 2**b** was simulated using the finite-element method (COMSOL). Table S2 summarizes the structures and corresponding materials with the relative permittivity ϵ, relative permeability μ, and electrical conductivity σ used for the calculations. The thin gold-patterned layer ($\sim$ 300 nm) was regarded as an infinitely thin perfect electric conductor (PEC) in the simulations. A 70-mm-radius sphere surrounding the coverslip was assumed to be a perfectly matched layer with an absorption constant of 10^{-6} at the sphere boundary.

TABLE S2. List of simulation conditions

Structures	Materials
Coverslip (thickness = 0.17 mm)	Borosilicate glass ($\epsilon = 4.6$, $\mu = 1.0$, $\sigma = 0.0$ S/m)
Thin gold layer (antenna)	Perfect electric conductor (PEC)
70-mm-radius sphere	Air ($\epsilon = 1.0, \mu = 1.0, \sigma = 0.0$ S/m)

S4. ODMR spectral analysis using a double-lorentzian function

The ODMR spectra were fitted to a double Lorentzian function comprising two Lorentzian functions (red and blue curves in Fig. S6**a**):

$$y(x) = y_0 + L_1(x) + L_2(x),$$
$$L_{1,2}(x) = \frac{2A_{1,2}}{\pi}\frac{w_{1,2}}{4(x - x_{1,2})^2 + w_{1,2}^2}, \quad \text{(S7)}$$

where y_0 is a common offset, and $A_{1,2}$, $w_{1,2}$, and $x_{1,2}$ are the peak area, line width, and peak position frequency for the left (index: 1) and right peaks (index: 2), respectively. The ODMR depth in Fig. 2**e** was obtained using $[y(x_1) + y(x_2)]/2$, and the depth in Fig. 2**e** was depicted as the "Norm. PL intensity". E and D shown in Figs. 2**e, f**, were calculated using $E = (x_2 - x_1)$ and $D = (x_1 + x_2)/2$, respectively. Note that the effect of the geomagnetic field (~ 0.5 G) [4] on E was insignificant because its potential variation of E is about 1.4 MHz which is comparable to the variations derived from the random NV quantization axes on the coverslip.

Figure S6**b** shows the amplitudes of $L_1(x_1)$ and $L_2(x_2)$ in the double Lorentzian fitting for all of the measured type-Ib and ^{12}C, N-NDs. The double Lorentzian model gives successful fitting results with comparable peak amplitudes for L_1 and L_2 under the applied boundary conditions (Fig. S6c). However, compared with those of type-Ib, ^{12}C, N-NDs sometimes exhibit asymmetric results with large differences between $L_1(x_1)$ and $L_2(x_2)$ ($\Delta L = |L_1(x_1) - L_2(x_2)|$), which might affect the results of E and D in Figs. 2**f,g**. The magnitude of ΔL does not affect the ODMR depth, as shown in Fig. 2**e**, because the contrast was obtained using $(y(x_1) + y(x_2))/2$, which only depends on the sum of $L_{1,2}(x)$.

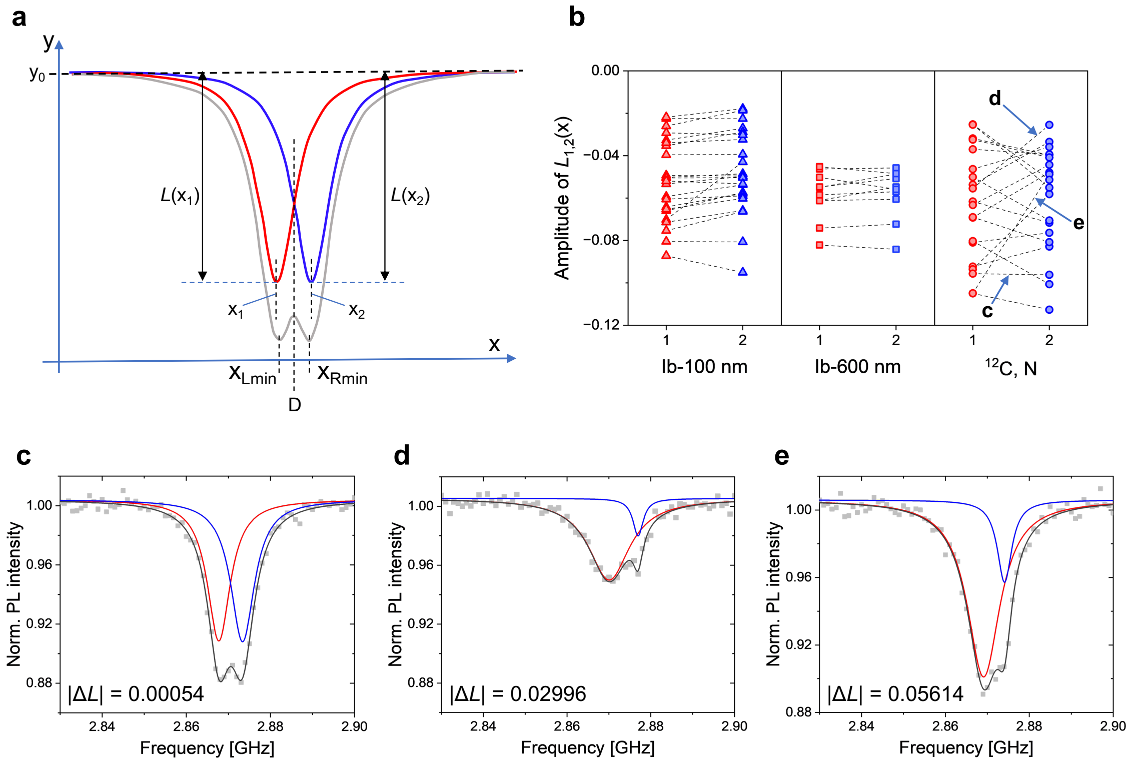

FIG. S6. (a) Schematic of the double-Lorentzian function fitted to the ODMR spectra. (b) Amplitudes of the fitted Lorentzian function ($L_1(x_1)$, $L_2(x_2)$) for the Ib-100, Ib-600, and ^{12}C, N-NDs. **c, d, e** indicate cases for Figs. S6**c, d, e**, respectively. (c–e) ODMR spectra with the fitting for the cases specified in Fig. S6**b**.

To evaluate this effect, we investigated individual fitting cases between NDs with small and large ΔL. We analyzed NDs with small and large ΔL values as representatives of the symmetric and asymmetric cases, respectively (Figs. S6**c**–**e**). To estimate the deviation of resulting from fitting destabilization (asymmetry), we compared E (D) values determined using the above defined fitting-based calculations ($E_{\rm fit}$ or $D_{\rm fit}$) with those by manually taking the frequency positions of the two peaks of the total double Lorentzian curves: $\Delta E_{\rm man} = x_{\rm Rmin} - x_{\rm Lmin}$ or $\Delta D_{\rm man} = x_{\rm Lmin} + x_{\rm Rmin}$,

where $x_{\mathrm{L(R)min}}$ indicates the minimum points of overall double Lorentzian shape (Gray curve in Fig. S6**a**). Table S3 summarizes these analyses for the three cases shown in Figs. S6**c**–**e**. $E_{\mathrm{fit}} - E_{\mathrm{man}}$ shows primarily positive values (0.74 and 0.71 MHz), and in the case of a large ΔL, it gives a more positive value (2.18 MHz), which means that the fitting destabilization tends to overestimate the E values compared to the apparent peak splitting of ODMR spectra. This result indicates that the observed small E values for the ^{12}C, N-NDs could not be attributed to underestimation from the fitting destabilization; rather, the E values are overestimated. Therefore, we conclude that the present fitting destabilization does not affect the observed tendency of small E values in the ^{12}C, N-NDs.

$D_{\mathrm{fit}} - D_{\mathrm{man}}$ can show both negative and positive values, and the magnitude can increase up to 0.00111 GHz, which is comparable to the observed difference between ^{12}C, N- and type-Ib NDs. Therefore, fitting destabilization may result in the observed tendency of relatively large D values in the ^{12}C, N-NDs. However, the ODMR spectra of ensemble NV exhibit a skew normal distribution shape [5]. This low-frequency skew is more prominent in type-Ib NDs than that in ^{12}C, N-NDs, and it pulls the fitting estimation of D toward the lower-frequency side for type-Ib NDs. Thus, the relatively large D values in the ^{12}C, N-NDs could be attributed to both fitting destabilization and intrinsic asymmetry of ODMR spectral shape in the type-Ib NDs.

TABLE S3. Fitting destabilization for E_{fit} and D_{fit}

Case	ΔL	$E_{\mathrm{fit}} - E_{\mathrm{man}}$ [MHz]	$D_{\mathrm{fit}} - D_{\mathrm{man}}$ [GHz]
Fig. S6**c**	0.00054	0.74	0.00010
Fig. S6**d**	0.02996	0.71	−0.00049
Fig. S6**e**	0.05614	2.18	0.00111

S5. Raman measurements

We investigated the lattice structures of type-Ib NDs (Ib-100, Ib-600) and ^{12}C, N-NDs using Raman spectroscopy. To detect the spectra, we made thick layers of the Ib-100 and Ib-600 NDs on p-type (100) silicon substrates by repeated drop-casting. For ^{12}C, N-NDs, a micron-sized ^{12}C, N-diamond ($\sim$ 100um in size) was placed on the silicon substrate. We used a commercially available Raman spectrometer (JASCO, NRS-5100NPS) with an excitation wavelength of 785 nm. Raman spectra for Ib-100 and Ib-600 NDs are shown in Figs. S7**a, b**, respectively, with typical diamond peaks observed at 1331 cm^{-1} for both. For ^{12}C, N-NDs, the diamond peak was observed at 1332 cm^{-1}, as shown in Fig. S7**c**.

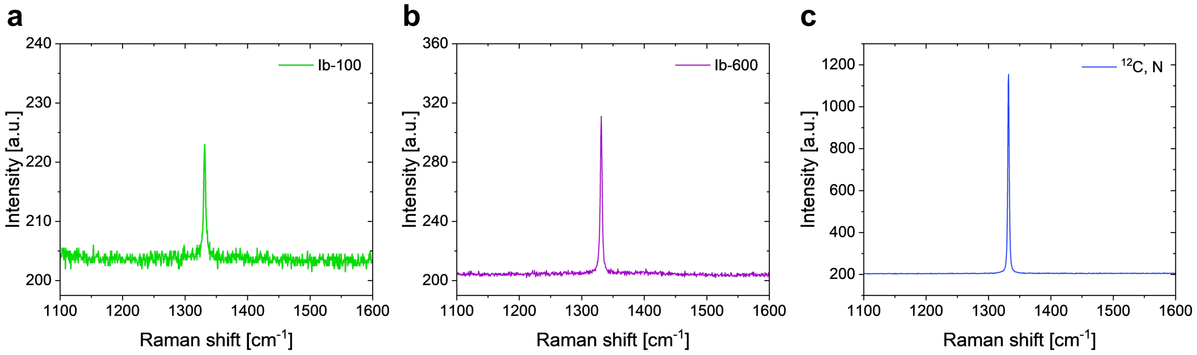

FIG. S7. Raman spectra for (a) Ib-100, (b) Ib-600 NDs, and (c) the micron-sized ^{12}C, N-diamond. The peak intensity varies depending on experimental parameters including the excitation position and sample thickness.

S6. Fitting procedure of the relaxation curves

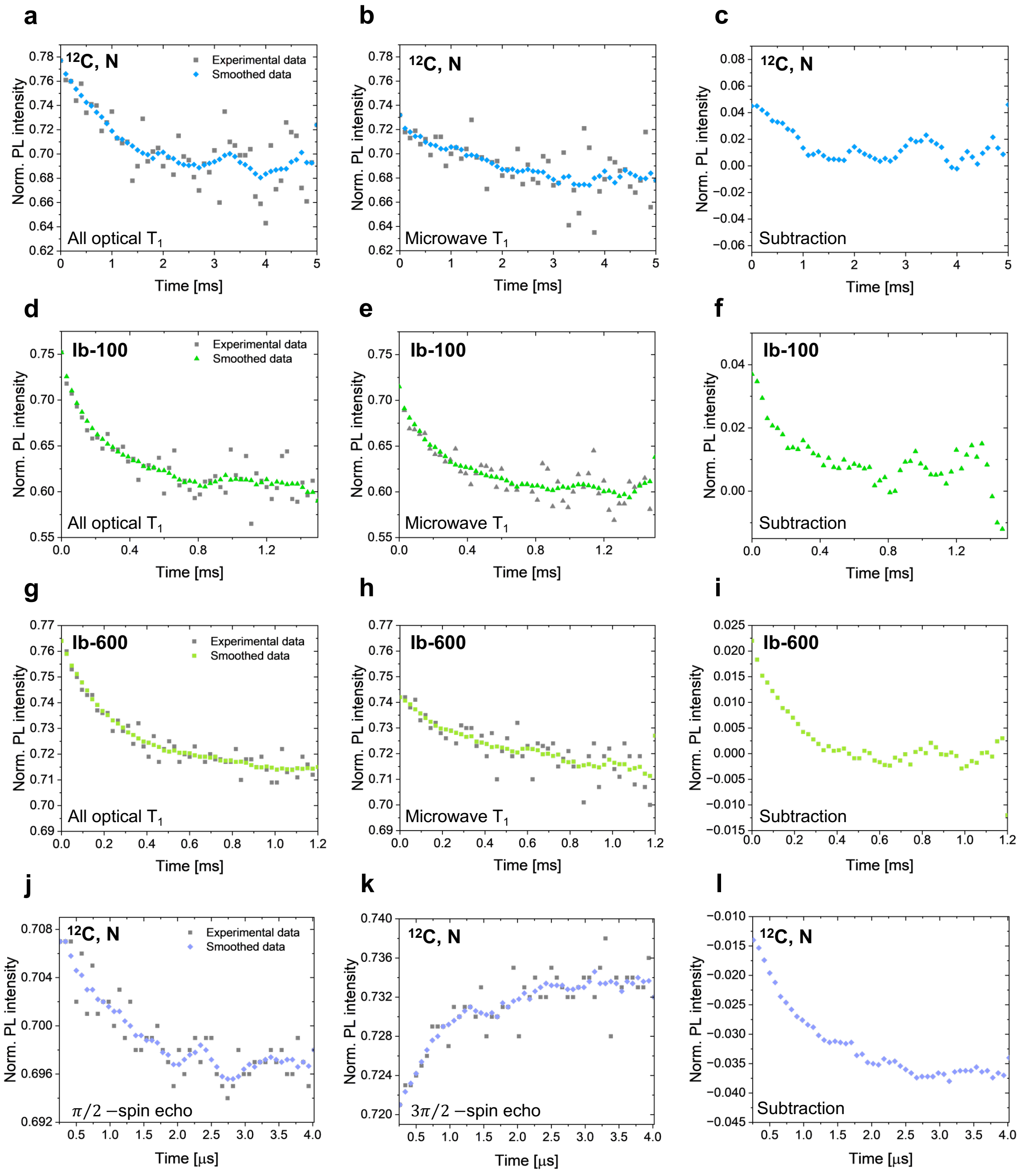

FIG. S8. (a) Representative profiles of all-optical T_1 and (b) microwave T_1 relaxometry for the ^{12}C, N-NDs. (c) The subtracted relaxation profiles. (Grey dots: experimental data, light blue dots: smoothed profiles by the moving average method). (d)–(f) Representative profiles of all-optical T_1 and microwave T_1 relaxometry, and subtracted relaxation for the Ib-100nm NDs. (Grey dots: experimental data, green dots: smoothed profiles by the moving average method). (g) Representative profiles for $\pi/2$-spin echo, and (h) $3\pi/2$-spin echo for the ^{12}C, N-NDs. (i) The subtracted profiles. (Grey dots: experimental data, lilac dot: smoothed profiles by the moving average method).

The raw T_1 relaxation profiles showed large noise, which destabilized the subsequent fitting processes. This noise must be numerically filtered to determine T_1 values. The profiles of microwave T_1 and all-optical T_1 were filtered by taking a moving average over nine data points from a total of 50 points and they were subtracted from each other, as shown in Figs. S8**a**–**f** (subtracting the microwave T_1 from the all-optical T_1 profile). The subtracted profiles were fitted using bi-exponential decay ($y = y_0 + A_1 \exp\left[-(\tau - t_0)/t_1\right] + A_2 \exp\left[-(\tau - t_0)/t_2\right]$), as defined in the Methods.

Similarly, the two spin-echo profiles were numerically filtered to remove noise by taking a moving average over five data points with a total of 50 points. The filtered profiles were subtracted to obtain the final T_2 profiles and a stretched exponential decay function $\exp\left((-\tau/T_2)^p\right)$ was fitted to determine T_2. The value of p can vary between 1.47 and 1.50 in the fitting, but we assumed $p = 1.5$ to determine T_2 for all T_2 profiles for the fitting consistency. Note that, in the above analysis of T_1 and T_2, the particle-based statistical error was adopted for the uncertainty of the mean relaxation times instead of the fitting error because fitting was applied to the numerically filtered data and the time step used in the measurements for the type-Ib and ^{12}C, N-NDs were different.

To determine T_2 of the Ib-100 NDs, we fitted the stretched exponential decay function to the $\pi/2$-spin echo profiles (Fig. S9**a**) because the $3\pi/2$-spin echo profiles did not differ from the $\pi/2$-spin echo (Fig. S9**b**), presumably because of the short T_2^* as compared with the microwave π pulse (300–400 ns). For the Ib-600 NDs, the different profiles between $\pi/2$-spin echo and $3\pi/2$-spin echo were obtained (Figs. S9**c, d**), but it was difficult to fit the subtracted profiles in a similar to the case of ^{12}C, N-NDs. Therefore, to determine T_2 of the Ib-600 NDs, we fitted the stretched exponential decay function to the $\pi/2$-spin echo profiles.

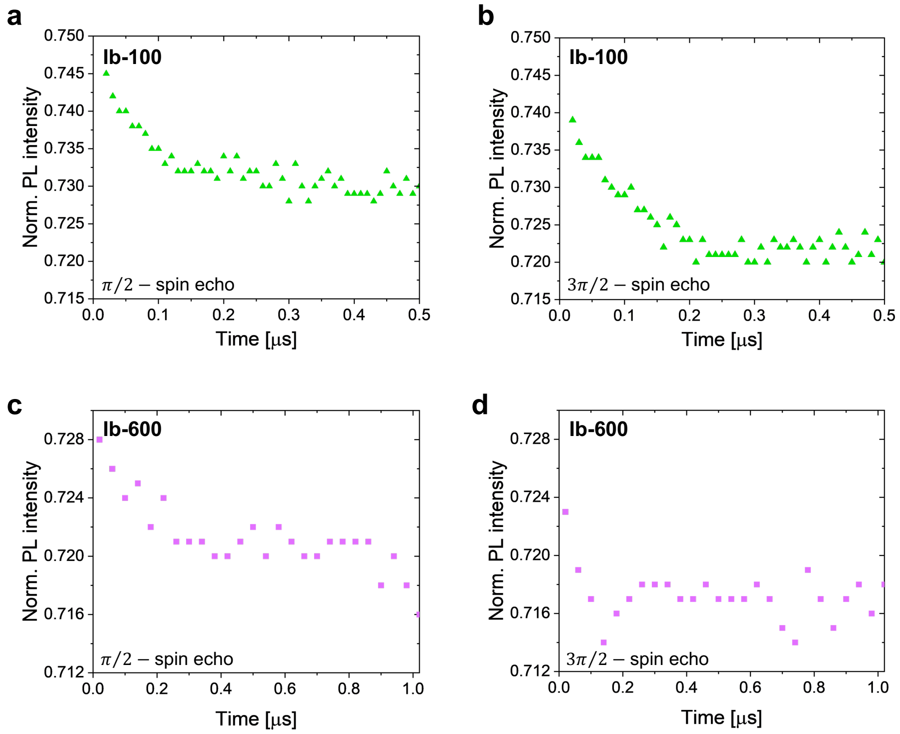

FIG. S9. (a) Representative profiles for $\pi/2$-spin echo and (b) $3\pi/2$-spin echo for the Ib-100 (green dots: experimental data). (c) Representative profiles for $\pi/2$-spin echo and (d) $3\pi/2$-spin echo for the Ib-600 (purple dots: experimental data).

S7. Cell culture protocol and other experimental results

Figure S10**a** shows a flow diagram of the cell culture protocol in our home-built multiwell device with a notch-shaped microwave antenna. Figure S10**b** shows the experimentally measured S_{11} and S_{21} spectra of the multiwell devices containing the cells, respectively. In addition to the ODMR experiments in the absence of an external magnetic field(Fig. 4**c**), using ND1 in the cells (Fig. 4**a**) under an external magnetic filed, we observed the CW-ODMR spectral shape changed in 4 min (Fig. S10**c**). This indicates the NV rotation owing to the rotational Brownian motion in a time interval of the experiments. This ND rotation is detrimental when measuring pulsed ODMR profiles under microwave excitation. NDs with 100–300 nm diameters exhibit 0.1–1 kHz (1–10 ms) as the characteristic frequency (time) of the rotational Brownian motion [6]. For example, in the $\pi/2$-spin echo measurements, a single operation of the sequence with a single echo time finished at approximately 2.0 µs at the longest, however, this spin operation accumulated over 500 ms. After 500 ms of integration, the measurements proceeded to the next sequence with the next echo time. By sweeping the echo time from 20–1020 ns at 40 ns intervals, we obtained a single dataset for the $\pi/2$-spin echo. Subsequently, the dataset was collected 15 times. The overall measurement time was 3–5 min. The time-varying NV orientation lead to apparent dephasing in the final T_2 profiles. Indeed, in the pulsed ODMR measurements on ND2, the Brownian motion interpreted the profiles of microwave T_1 and two spin-echo sequences (Figs. S10**d, e**). All-optical T_1 relaxometry is an exception, which utilizes time-dependent spin polarization after optical initialization [7] (Fig. S10**d**, top panel). The profile exhibited a millisecond-scale relaxation. Double exponential fitting gives a value of $T_1 = 0.87$ ms, which is six times larger than the mean T_1 value of type-Ib NDs. In contrast, the T_2 relaxation profiles exhibit a substantial shortening of T_2 owing to the Brownian motion. Figure S10**e** shows the relaxation profile of the $\pi/2$- and $3\pi/2$-spin-echo sequence. Single-exponential fitting on the $\pi/2$-spin-echo profile yields $T_2 = 135$ ns, which is one order of magnitude shorter than the mean T_2 value determined in main text.

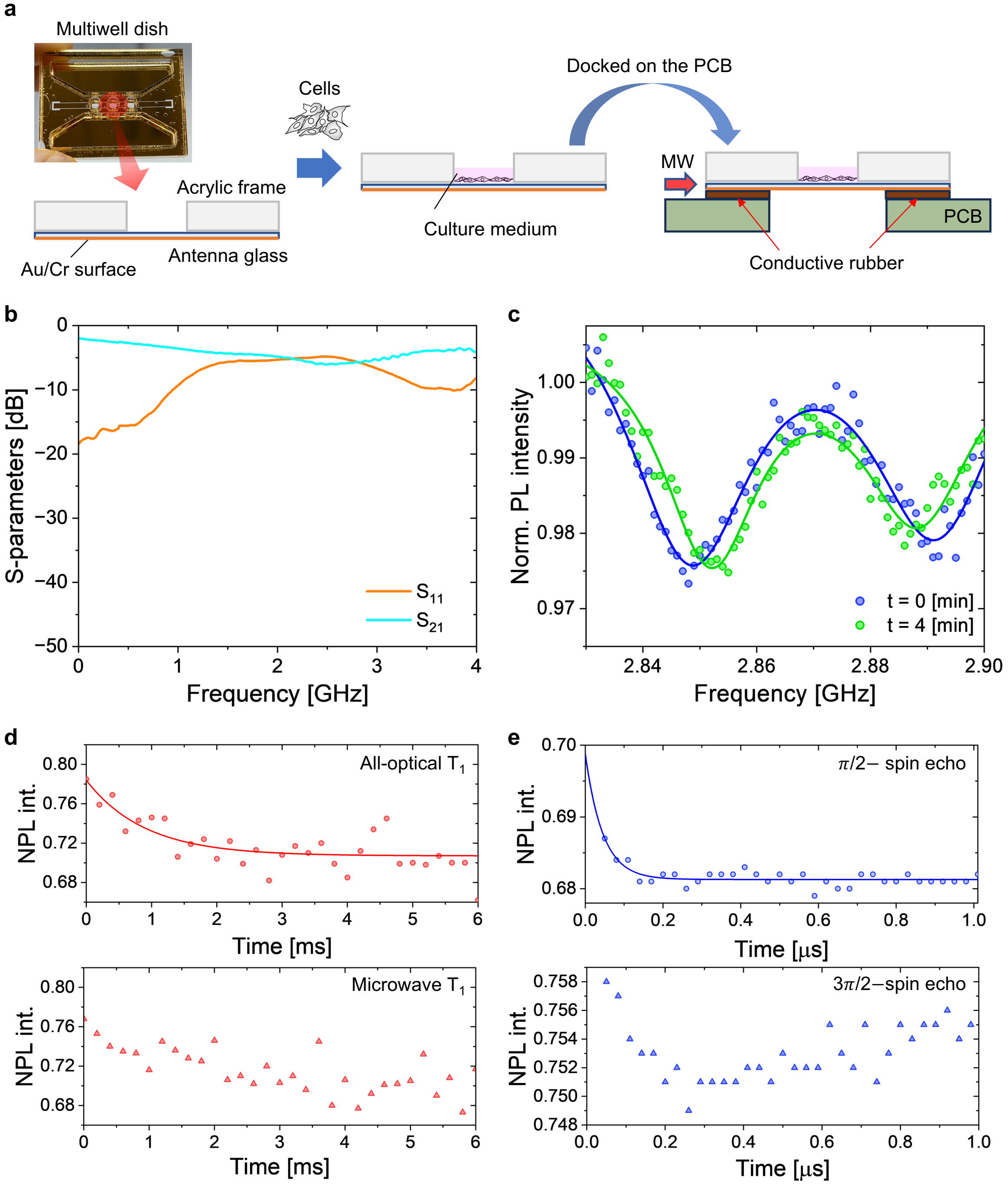

FIG. S10. (a) Flow diagram for the device assembly and ODMR measurements using cells and (b) experimentally measured S_{11} and S_{21} spectra of the device. (c) Split ODMR spectra inside cells under an external magnetic field (blue dots) and the subsequent ODMR spectra of the same NDs in 4 min (green dots). (d) Microwave T_1 and (e) $3\pi/2$-spin echo for ND2 inside the cell in Fig. 4**a**. (d) All-optical T_1 relaxometry and (e) $\pi/2$-spin echo profiles for ND2 in an external magnetic field.

S8. A series of experiments related to thermal echo measurements

Figure S11**a** shows the CW-ODMR spectrum for a representative ^{12}C, N-ND in an external magnetic field. Using the same ^{12}C, N-ND, we subsequently performed pulsed experiments including Rabi, spin-echo before CPMG, and TE measurements. Figures S11**a, b** show representative profiles of Rabi oscillation and spin echo, respectively.

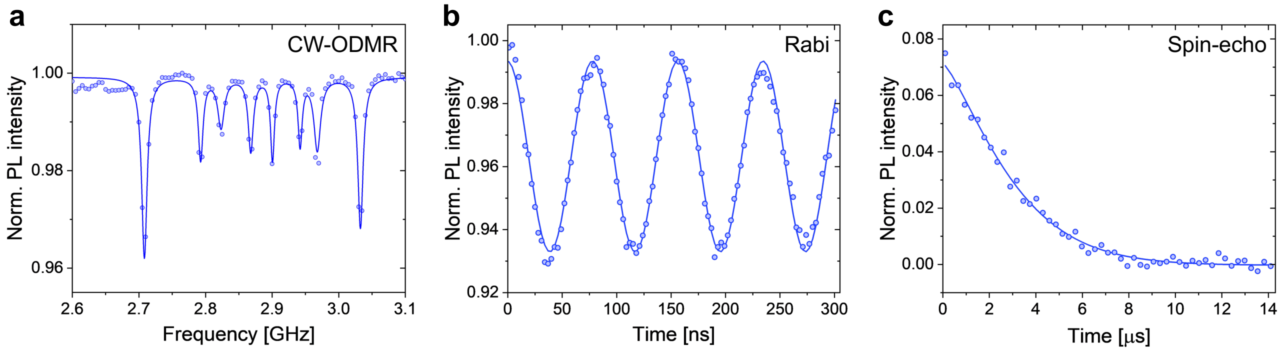

FIG. S11. (a) ODMR spectrum of representative ^{12}C, N-ND in external magnetic field, indicating eight resonance frequencies corresponding to the four NV quantization axes. (b) Representative profiles of the Rabi oscillation and (c) spin echo for representative ^{12}C, N-ND ($T_2 = 3.32\,\mu s$).

S9. Determination of nitrogen concentration for type-Ib NDs from FTIR spectrum

SIMS is widely used to determine the nitrogen impurity concentration in bulk diamonds [8]. However, in our case, the crystal size of the type-Ib NDs was too small to be measured using SIMS. EPR spectroscopy can quantify the nitrogen concentration in NDs [9]. However, it requires gram level amount of NDs, which is prohibitively high for the present case. Therefore, we employed FTIR spectroscopy to analyze the concentration of nitrogen in the conventional type-Ib NDs. This measurement was carried out using a Jasco FTIR6200-IRT7000 micro Fourier-transform spectrometer with a KBr/Ge beam splitter and ceramic light source (see Methods). Figure S12**a** shows a diamond micro-crystal taken from the same product line of the type-Ib NDs used in this study. Figure S12**b** shows a typical FTIR spectrum of these diamond micro-crystals. From this spectrum, we calculated $(\mu_{1130\mathrm{cm}^{-1}}/\mu_{2120\mathrm{cm}^{-1}}) \times 5.5 \times 25$ to determine the nitrogen concentration [N] (ppm) [10]. The [N] of these micro-crystals was estimated to 300–540 ppm, which was obtained by measuring five crystals in total. The variation in [N] may be caused by several factors, including particulate inhomogeneity, imperfect perpendicular incidence of the NIR beams on the diamond surface because of the unavoidable angle created on the copper mesh during the FTIR measurements.

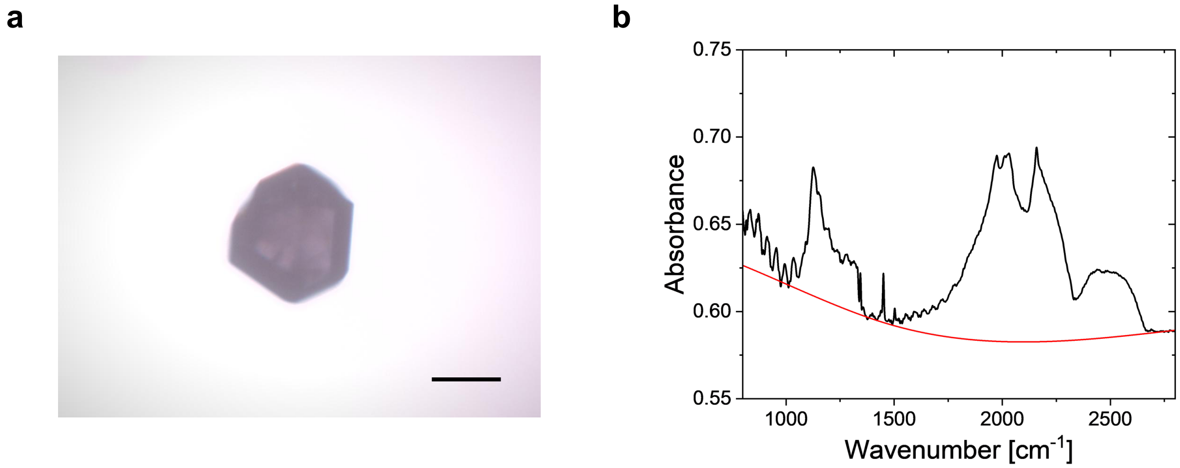

FIG. S12. (a) Microscope image of type-Ib micro-sized diamond in the FTIR measurements. Scale bar: 100 µm. (b) Representative FTIR spectrum for type-Ib diamonds. Red curve: base line.

S10. Rabi oscillation comparison and Ramsey experiments

The inhomogeneity in the NV spin environment diminishes Rabi contrast (Fig. S13**a**), which makes the subsequent pulsed ODMR measurements challenging particularly for the type-Ib NDs. This inhomogeneity can be evaluated via T_2^* measured by Ramsey experiments (Fig. S13**b**). Ramsey oscillations for a certain ^{12}C, N-ND is shown in Fig. S13**c**.

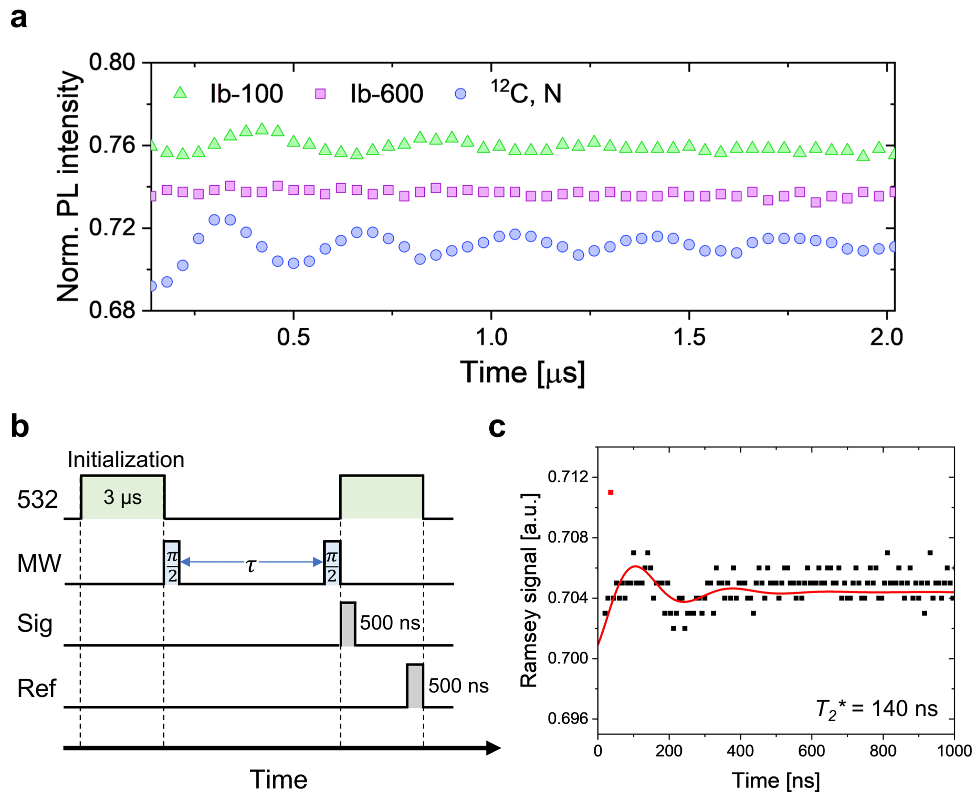

FIG. S13. (a) Comparison of Rabi oscillations between the different types of NDs at the same microwave power. Green triangle: Ib-100, purple square: Ib-600, blue circle: ^{12}C, N-NDs. In these measurements, we accumulated the data 20 times with the same measurement time step. (b) Schematic sequences of the Ramsey measurement. (c) Ramsey oscillation of a ^{12}C, N-ND, where the data was accumulated 20 times. The observed signal (plot data) is fitted using the sine-damping equation (red line). A red point contained one-time noise spike and excluded from the fitting.

[1] J. Canet-Ferrer, E. Coronado, A. Forment-Aliaga, and E. Pinilla-Cienfuegos, "Correction of the tip convolution effects in the imaging of nanostructures studied through scanning force microscopy," Nanotechnology **25**, 395703 (2014).

[2] S. Eldemrdash, G. Thalassinos, A. Alzahrani, Q. Sun, E. Walsh, E. Grant, H. Abe, T. L. Greaves, T. Ohshima, P. Cigler, P. Matějíček, D. A. Simpson, A. D. Greentree, G. Bryant, B. C. Gibson, and P. Reineck, "Fluorescent HPHT nanodiamonds have disk-and rod-like shapes," Carbon **206**, 268–276 (2023).

[3] K. Oshimi, Y. Nishimura, T. Matsubara, M. Tanaka, E. Shikoh, L. Zhao, Y. Zou, N. Komatsu, Y. Ikado, Y. Takezawa, E. Kage-Nakadai, Y. Izutsu, K. Yoshizato, S. Morita, M. Tokunaga, H. Yukawa, Y. Baba, Y. Teki, and M. Fujiwara, "Glass-patternable notch-shaped microwave architecture for on-chip spin detection in biological samples," Lab Chip **22**, 2519–2530 (2022).

[4] A. Kuwahata, T. Kitaizumi, K. Saichi, T. Sato, R. Igarashi, T. Ohshima, Y. Masuyama, T. Iwasaki, M. Hatano, F. Jelezko, M. Kusakabe, T. Yatsui, and M. Sekino, "Magnetometer with nitrogen-vacancy center in a bulk diamond for detecting magnetic nanoparticles in biomedical applications," Sci. Rep. **10**, 2483 (2020).

[5] G. J. Abrahams, E. Ellul, I. O. Robertson, A. Khalid, A. D. Greentree, B. C. Gibson, and J. P. Tetienne, "Handheld device for noncontact thermometry via optically detected magnetic resonance of proximate diamond sensors," Phys. Rev. Appl. **19**, 054076 (2023).

[6] M. Fujiwara, Y. Shikano, R. Tsukahara, S. Shikata, and H. Hashimoto, "Observation of the linewidth broadening of single spins in diamond nanoparticles in aqueous fluid and its relation to the rotational brownian motion," Sci. Rep. **8**, 14773

(2018).

[7] A. Sigaeva, H. Shirzad, F. P. Martinez, A. C. Nusantara, N. Mougios, M. Chipaux, and R. Schirhagl, "Diamond-based nanoscale quantum relaxometry for sensing free radical production in cells," Small **18**, 2105750 (2022).

[8] K. Ikeda and H. Sumiya, "Optical properties of ultrapure nano-polycrystalline diamond," Jpn. J. Appl. Phys. **55**, 120306 (2016).

[9] T. Teraji and C. Shinei, "Nitrogen-related point defects in homoepitaxial diamond (001) freestanding single crystals," J. Appl. Phys. **133**, 165101 (2023).

[10] Z. Z. Liang, X. Jia, H. A. Ma, C. Y. Zang, P. W. Zhu, Q. F. Guan, and H. Kanda, "Synthesis of HPHT diamond containing high concentrations of nitrogen impurities using NaN_3 as dopant in metal-carbon system," Diam. Relat. Mater. **14**, 1932–1935 (2005).
